# An Evaluation of the Potential of Essential Oils against SARS-CoV-2 from In Silico Studies through the Systematic Review Using a Chemometric Approach

**DOI:** 10.3390/ph14111138

**Published:** 2021-11-10

**Authors:** Luiz Torres Neto, Maria Lúcia Guerra Monteiro, Diego Galvan, Carlos Adam Conte-Junior

**Affiliations:** 1 COVID-19 Research Group, Center for Food Analysis (NAL), Technological Development Support Laboratory (LADETEC), Cidade Universitária, Rio de Janeiro 21941-598, Brazil; luiztorresneto@ufrj.br (L.T.N.); marialuciaguerra@yahoo.com.br (M.L.G.M.); diegogalvann@gmail.com (D.G.); 2 Laboratory of Advanced Analysis in Biochemistry and Molecular Biology (LAABBM), Department of Biochemistry, Federal University of Rio de Janeiro (UFRJ), Cidade Universitária, Rio de Janeiro 21941-909, Brazil; 3 Graduate Program in Food Science (PPGCAL), Institute of Chemistry (IQ), Federal University of Rio de Janeiro (UFRJ), Cidade Universitária, Rio de Janeiro 21941-909, Brazil; 4 Institute of Chemistry, Federal University of Rio de Janeiro, Avenida Athos da Silveira Ramos, n. 149, Bloco A, 5° Andar, Rio de Janeiro 21941-909, Brazil; 5 Graduate Program in Veterinary Hygiene (PPGHV), Faculty of Veterinary Medicine, Fluminense Federal University (UFF), Vital Brazil Filho, Niterói 24220-000, Brazil; 6 Graduate Program in Sanitary Surveillance (PPGVS), National Institute of Health Quality Control (INCQS), Oswaldo Cruz Foundation (FIOCRUZ), Rio de Janeiro 21040-900, Brazil

**Keywords:** COVID-19, plant species, volatile compound, antiviral activity, hierarchical cluster analysis (HCA)

## Abstract

Essential oils (EOs) and their compounds have attracted particular attention for their reported beneficial properties, especially their antiviral potential. However, data regarding their anti-SARS-CoV-2 potential are scarce in the literature. Thus, this study aimed to identify the most promising EO compounds against SARS-CoV-2 based on their physicochemical, pharmacokinetic, and toxicity properties. A systematic literature search retrieved 1669 articles; 40 met the eligibility criteria, and 35 were eligible for analysis. These studies resulted in 465 EO compounds evaluated against 11 human and/or SARS-CoV-2 target proteins. Ninety-four EO compounds and seven reference drugs were clustered by the highest predicted binding affinity. Furthermore, 41 EO compounds showed suitable drug-likeness and bioactivity score indices (≥0.67). Among these EO compounds, 15 were considered the most promising against SARS-CoV-2 with the ADME/T index ranging from 0.86 to 0.81. Some plant species were identified as EO potential sources with anti-SARS-CoV-2 activity, such as *Melissa officinalis* Arcang, *Zataria multiflora* Boiss, *Eugenia brasiliensis* Cambess, *Zingiber zerumbet* Triboun & K.Larsen, *Cedrus libani* A.Rich, and *Vetiveria zizanoides* Nash. Our work can help fill the gap in the literature and guide further in vitro and in vivo studies, intending to optimize the finding of effective EOs against COVID-19.

## 1. Introduction

Essential oils (EOs) are volatile liquids extracted from whole or parts of aromatic plants such as bark, fruits, flowers, and leaves [1]. These compounds have a broad biological spectrum with antimicrobial, antifungal, insecticide, anti-inflammatory, antioxidant, anticarcinogenic, and antiviral actions [2]. Accordingly, interest in EOs has been increasing in different fields, such as food, pharmaceutical, cosmetic, and medical applications [2,3]. In this way, the biological potential of these compounds can be valuable for combating the COVID-19 pandemic.

The disease caused by SARS-CoV-2 (COVID-19) is still causing infection and mortality worldwide. More than 245 million people have been infected worldwide, with almost five million deaths by mid-October 2021 [4]. The large influx of patients challenged the health systems of several countries. Thus, numerous public health measures are still needed to contain virus transmission [5], mainly by new mutations and expected variants [6], despite the large vaccination campaigns already started in several countries [7].

The symptoms of SARS-CoV-2 are similar to severe acute respiratory syndrome coronavirus (SARS-CoV) and Middle Eastern respiratory syndrome coronavirus (MERS-CoV), which have an incubation period of two weeks for express signs such as fever, cough, dyspnea, and lung damage [8]. The infection process occurs through a specific receptor called angiotensin-converting enzyme-2 (ACE2), which allows the virus entry into the host cell through the active receptor-binding domain (RBD) in the spike protein (S^pro^) found on the virus surface [9,10]. Recent studies show that blocking ACE2 and the S^pro^ is one possible way of preventing SARS-CoV-2 from entering the target cell. Thus, compounds with the potential to inhibit one of these proteins would be critical for identifying new anti-SARS-CoV-2 compounds [9,11].

The antiviral potential of EOs is attributed to their composition of mono- and sesquiterpenes hydrocarbons. These compounds are promising in prospecting studies of antiviral molecules [12]. Furthermore, the antiviral action of some EOs is already known against viruses such as human herpes virus (HSV), influenza A virus (H1N1), avian influenza A virus (H5N1), Zika virus, and human immunodeficiency virus (HIV), among others [13]. In association with the increasing demand for natural and safe products, these facts make EOs a potential alternative to aid in combating COVID-19.

Review studies have assumed that EOs have antiviral potential against SARS-CoV-2 due to their known action against several other viruses and for managing symptoms in patients with COVID-19 [14,15,16,17]. Despite that, there is little data gathered regarding the active potential of EOs against the SARS-CoV-2 virus. In this context, the present study aimed to identify the most promising EO compounds and potential EO sources against COVID-19 based on their affinity by target proteins, drug-likeness, bioactivity score, and ADME/T through a systematic review and chemometric analysis.

## 2. Material and Methods

**Figure 1:**
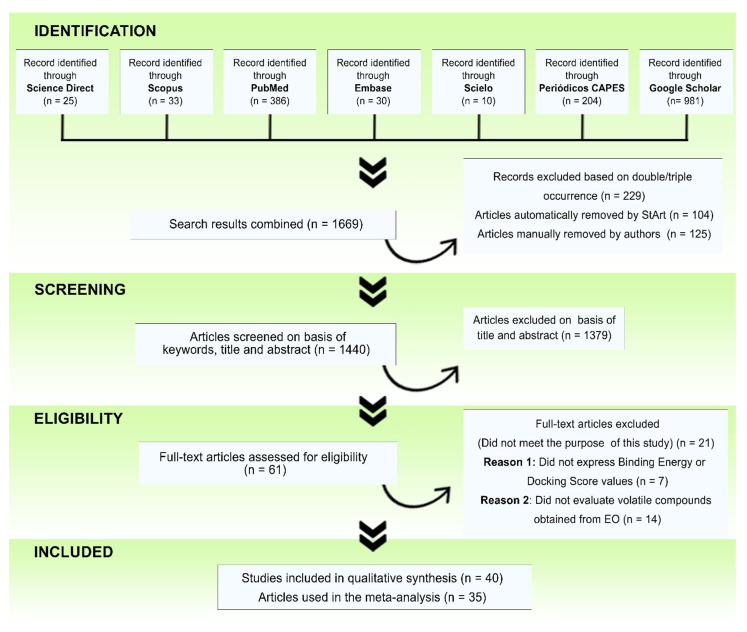
Flow diagram displaying the results of the systematic literature search about the antiviral potential of compounds from essential oils (EOs) against SARS-CoV-2 between 2019 and 2021 through Preferred Reporting Items for Systematic Review and Meta-Analyses (PRISMA) and StArt software [18,19].

In this study, we systematically retrieved data available in online databases on studies relating to the antiviral potential of compounds from EOs against SARS-CoV-2 following the Preferred Reporting Items for Systematic Review and Meta-Analyses (PRISMA; http://www.prisma-statement.org/PRISMAStatement/; accessed on 15 August 2021) guidelines (see Supplementary File 1 and Figure 1) [18], and StArt software [19] was used.

### 2.1. Focus Questions

The focus issue was determined according to the population, intervention, comparison, and outcome (PICO) method. The research questions were based on the following form:

(P) Do EO compounds have potential action against SARS-CoV-2?
(I) Which EO compounds have been studied for action against SARS-CoV-2?
(C) What are the possible mechanisms of action and the most promising EO compounds against SARS-CoV-2?
(O) What are the botanical genera with the most expressive action against SARS-CoV-2?

### 2.2. Information Source

An exploratory analysis without a specific period was previously performed on Google Scholar using strings that summarize the issues raised: (COVID-19 OR Coronavirus OR SARS-CoV-2 OR “COVID-19 Pandemic”) AND “Essential oil” AND (Viral OR Antiviral OR “Viral action” OR “Antiviral activity” OR “Antiviral agent”). This step was performed to identify the main words and their synonyms used in the title, abstract, and keywords of articles related to the subject of this study for subsequent building of the search components (SC).

The search was based on online databases, using PubMed, Scopus, Embase, Science Direct, Google Scholar, SciELO, and the Higher-Level Personnel Improvement Coordination Gateway (Periódicos CAPES). Periódicos CAPES is a vast virtual library that allows simultaneous access to PubMed, Web of Science, Scopus, Springer Link, Wiley Online Library, Taylor & Francis Online, and Embase. The entire search process in the databases was carried out between 4 and 6 May 2021, identifying studies published between 2019 and 2021, according to the following search strings:

Search component 1 (SC1): SARS-CoV-2 OR COVID-19 OR 2019-nCoV OR Coronavirus
Search component 2 (SC2): “Essential Oil” OR “Volatile Oil”
Search component 3 (SC3): Antiviral OR Virucide

After retrieving the search component results, the Boolean operator “AND” combined SC1, SC2, and SC3.

### 2.3. Inclusion/Exclusion Criteria

All screening processes were performed by L.T.N. and M.L.G.M. The replicated studies were automatically eliminated by the StArt software from each database, and a manual exclusion was also performed. Then, the articles were screened based on the title, abstracts, and keywords according to the eligibility criteria. The inclusion criteria used in this study were:

(i). Articles published in the English language;
(ii). Peer-reviewed original articles and preprints;
(iii). Studies evaluating the activity of compounds from EOs concerning their anti-SARS-CoV-2 action in silico and/or in vitro.

The exclusion criteria used were:

(i). Abstracts, books, conference articles, editorials, letters, reviews, meta-analyses, presentations, reviews, and doctoral theses;
(ii). Articles evaluating the antiviral action of compounds from EOs on other viruses;
(iii). Articles that evaluated the antiviral action of other compounds against SARS-CoV-2;
(iv). Studies evaluating the effect of EO compounds against SARS-CoV-2 but without considering the target parameters of this study.

Articles that generated doubts regarding the eligibility criteria were considered for a full reading.

### 2.4. Evaluation of Articles, Data Extraction, and Analyses

After reading the articles in full, 40 articles were eligible for the present study (Figure 1), of which 92.5% (*n* = 37) were molecular docking (MD) studies of EO compounds against target proteins that participate in the process of infection and viral replication of SARS-CoV-2 in the human cell [3,9,20,21,22,23,24,25,26,27,28,29,30,31,32,33,34,35,36,37,38,39,40,41,42,43,44,45,46,47,48,49,50,51,52,53,54]. Only 7.5% (*n* = 3) were in vitro studies [55,56,57]. Due to the low number of in vitro studies, they were not included in the statistical analysis. However, they were systematically demonstrated to reinforce our findings in a complementary way. Data extracted from all eligible in silico studies were sent to an MS Excel spreadsheet, following the criteria: name of the first author followed by “et al”., year of publication, EO source, evaluated EO compound (including reference drugs), human and/or SARS-CoV-2 protein targets, binding energy (BE), and docking score (DS) values. When applicable, BE and DS values were converted to Kcal/mol.

The data extracted from the MD studies generated a set of 1271 data with a total of 465 EO compounds, 13 reference drugs, and 11 target proteins. The mean was calculated for the BE and DS values of the same compounds evaluated against the same target proteins, and it was considered in our study when the coefficient of variation (CV) was less than 8. Otherwise, the average was considered an outlier. Furthermore, some BE and DS values of different compounds in the same protein were considered outliers within our data set [26,38].

After that, the BE and DS values were submitted to a hierarchical cluster analysis (HCA) together with a heat map graph (Section 2.6). This analysis aimed to aggregate the compounds with more significant similarities into clusters, identifying those with spontaneous binding potential (lower BE and DS values) in each target protein [58,59]. In other words, the most promising EO compounds against SARS-CoV-2 based on BE and DS values were identified for further characterization regarding their physicochemical, pharmacokinetic, and toxicity properties.

#### 2.4.1. Drug-Likeness Prediction

Lipinski’s rule of five (RO5) is a significant parameter for describing the molecular properties of compounds for estimating important pharmacokinetic parameters. Ghose’s rule is a filter designed to improve drug similarity predictions [60], and Muegge’s rule is based on the presence of structural fragments typically found in drugs [61]. All EO compounds and reference drugs were tested for Lipinski’s rule with the web-based tool Molinspiration (https://www.molinspiration.com/; accessed on 15 August 2021) [62], and Ghose’s and Muegge’s rules through the SwissADME server (http://www.swissadme.ch/; accessed on 15 August 2021) [63].

The promising compounds are those with no violations or at most one violation of these rules. Therefore, using Lipinski’s, Ghose’s, and Muegge’s rules violation data, a drug-likeness index (DLI) was created by assigning values of 1 (violation = 0) and 0 (violation ≥ 1) through the formula: DLI = (Lipinski + Ghose + Muegge)/(*n*_total rules_). The DLI indices were subjected to HCA with a heat map graph, in which promising EO compounds were those clustered by a DLI equal to or greater than 0.67.

#### 2.4.2. Bioactivity Score Prediction

The bioactivity score of each EO compound and the reference drugs was predicted using the Molinspiration tool. This allows for the identification of the bioactivity of drug candidates in some human receptors [64], such as binding to the G protein-coupled receptor (GPCR) ligand and nuclear receptor ligand, ion channel modulation, kinase inhibition, protease inhibition, and enzyme activity inhibition [62]. Using the data generated by the tool, scores were assigned to the activity of each compound: 1 (inactive > −0.50), 2 (moderately active from −0.50 to 0.00), and 3 (active > 0.00), according to previously defined criteria [65]. The scores were used to calculate the bioactivity score index (BSI): BSI = (GPCRs + nuclear receptors + ion channels + kinases + proteases + enzymes)/(n_sum of scores_). The BSI indices were subjected to HCA with a heat map graph, considering values equal to or greater than 0.67 as promising.

#### 2.4.3. ADME/T

The compounds with the highest DLI and BSI scores were evaluated for pharmacokinetic and toxicity properties (ADME/T). First, 22 parameters of the absorption, distribution, metabolism, excretion, and toxicity of the compounds were evaluated by the ADMETlab server (http://admet.scbdd.com/; accessed on 15 August 2021). Categorical and numeric values were considered “positive/beneficial” (green) or “negative/harmful” (red) based on the interpretation provided by the server (see Supplementary File 2) [63]. Next, a score was assigned to each compound as follows: 1 for green properties and 0 for red properties [66]. For calculating the ADME/T index, data were then converted to values from 0 to 1 as in Section 2.4.1 and Section 2.4.2. Then, the ADME/T indices were subjected to HCA with a heat map graph. Values close to 0 were considered the worst, while those close to 1 were considered the most promising.

### 2.5. Risk of Bias Assessment

The possible sources of bias include the study exclusion/inclusion criteria, chosen database, language, different MD programs used in different studies, and article type selected for this study. The quality of the studies was assessed based on articles that were published in peer-reviewed journals [67].

### 2.6. Visual and Statistical Analysis

A word cloud was generated in the RStudio software “word clouds” package to visualize the most frequently studied EO compounds against SARS-CoV-2 [68]. HCAs with heat map graphs were performed using the toolbox “HeatMapDendrogram” in the OriginPro software (OriginLab Corporation). For the HCA setup, the Euclidean distance and Ward’s linkage algorithm were used to hierarchically group the EO compounds and reference drugs into clusters according to their similarity concerning each evaluated parameter: the BE/DS regarding specific target proteins, DLI, BSI, and ADME/T index [2,69].

## 3. Results and Discussion

### 3.1. Main EO Compounds Evaluated against SARS-CoV-2

In the search for new molecules with the potential to inhibit the coronavirus, studies have focused on plant species already known for their rich composition of bioactive compounds with the potential to impair viral replication or support the treatment of some symptoms of COVID-19, specifically in the reduction of the self-perception of dyspnea and the inhibition of pulmonary ventilation. It has been proposed that many EO compounds reach the respiratory tract by inhalation, mainly due to their volatility and successful use in treating other respiratory tract infections. Consequently, EOs can contribute positively to symptoms such as cough, mucus, nasal congestion, runny nose, or sore throat [14,70].

**Figure 2:**
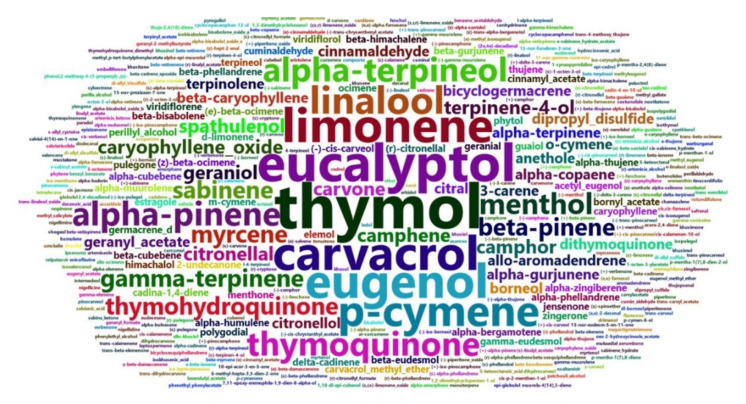
Word cloud identifying the most evaluated essential oil (EO) compounds in molecular docking studies against SARS-CoV-2.

A word cloud is a visual representation of the frequency and number of words most present in a dataset [68]. Figure 2 illustrates the main EO compounds evaluated in the in silico studies against SARS-CoV-2. Compounds such as thymol, eucalyptol (1,8-cineole), carvacrol, limonene, camphene, thymoquinone, and carvone, among others, were widely evaluated against different target proteins of the coronavirus. The most frequent compounds are present mainly in EOs from the *Nigella sativa* Boiss. species and genus *Eucalyptus*.

The EO from *Eucalyptus globulus* Labill. has eucalyptol as its major compound (52.47%) [3]. It is an antimicrobial and anti-inflammatory agent, which may play an important role in clinical outcomes in patients with COVID-19 [14,71]. In the study by Li et al. [72], eucalyptol showed protection against influenza A virus (IFV) in mice, attenuating the inflammatory responses. Limonene is also present in this EO, presenting anti-inflammatory and immunomodulatory potential, and it is being considered as a candidate for COVID-19 treatment [73]. Both eucalyptol and limonene are also present in EOs such as *Pelargonium graveolens* L’Hér. and *Citrus limon* Osbeck, which showed an inhibitory effect against ACE2 in epithelial cells [56].

Another plant frequently evaluated in studies with anti-SARS-CoV-2 potential (*n* = 8) was black cumin (*N. sativa*), which is composed mainly of trans-anethole, p-cymene, limonene, carvone, α-thujene, thymoquinone (TQ), thymohydroquinone (THQ), dithymoquinone, carvacrol, and β-pinene [74,75]. This composition gives it medicinal potential against neurological and mental diseases, cardiovascular disorders, cancer, diabetes, and inflammatory and viral diseases [76]. There are few reports in the literature about the antiviral action of EOs from *N. sativa*. However, a study by Labib and Sohrab [77] revealed that a plant oil containing thymoquinone showed a reduction in viral infection in a model of murine cytomegalovirus (MCMV).

Thymol, carvacrol, and eugenol are compounds already known for their biological potential. Previously, carvacrol exhibited a significant reduction in lung inflammation in mice with emphysema [78]. In the study by Vimalanathan and Hudson [79], eugenol vapor demonstrated rapid action against the IFV. In the same way, thyme EO, which has a high amount of thymol in its composition, showed antiviral activity against herpes simplex virus type 1 (HSV-1), human rhinoviruses (HRV), and the IFV [80], justifying the interest in evaluating the effectiveness of these compounds against SARS-CoV-2.

### 3.2. Selection of EO Compounds

The binding energy (BE) and docking score (DS) through MD analysis are frequently used as screening parameters since they indicate the stability of the interaction between the compound and target protein considering their binding sites [49,50]. Therefore, the promising EO compounds and the reference drugs were those grouped in the cluster with the highest predicted affinity based on the BE and DS values for each evaluated human and SARS-CoV-2 target protein (Supplementary File 3).

In total, 22 EO compounds were selected for the main protease (M^pro^/3CL^pro^) (BE = 8; DS = 14), 46 for the spike protein (S^pro^) (BE = 36; DS = 13), 13 for ACE2 (BE = 7; DS = 6), and 6 for the ACE2-S^pro^ complex (BE = 4; DS = 2) (see Supplementary File 3 (Figures S1–S4)). The compounds clustered by the lowest BE and DS values were also selected for the enzyme transmembrane protease serine type 2 (TMPRSS2) (BE = 4), nonstructural protein 9 RNA binding protein (BE = 3), replicase polyprotein (BE = 3), cathepsin B (CatB) (BE = 2), cathepsin L (CatL) (BE = 5), RNA-dependent RNA polymerase (DS = 6), endoribonuclease (DS = 21), and ADP ribose phosphatase (DS = 16) (see Supplementary File 3 (Figures S5–S11)). In the end, 101 different compounds were screened, of which 94 were EO compounds and 7 were reference drugs (artemisinin, camostat, remdesivir, arbidol, chloroquine, favipiravir, and hydroxychloroquine).

Among the screened compounds, some of them are present in EOs already known for their antiviral potential. β-caryophyllene, present in the EO of *Zataria multiflora* Boiss., had a high binding affinity with M^pro^ (DS: −7.7 Kcal/mol), showing a high similarity with some reference drugs (remdesivir, favipiravir, and hydroxychloroquine). This sesquiterpene showed a selectivity index (SI) of 55.4 with an IC50 of 0.003%, contributing to the antiviral activity against HSV-1 [81]. The EO from *Z. multiflora* also contains thymol (−6.9 Kcal/mol) and caryophyllene oxide (−7 Kcal/mol), which exhibited a high binding affinity to S^pro^, similar to artemisinin, a reference drug. Furthermore, caryophyllene oxide showed similarity to remdesivir against RP1a and nonstructural protein 9 RNA-binding protein (Supplementary File 3). A previous study revealed that isolated thymol compounds showed 96% inhibition against the SARS-CoV-2 virus in the Vero E6 cell line [55].

The EO of *Eucalyptus bicostata* Maiden, Blakely & Simmonds evaluated against coxsackievirus B3 showed antiviral action at 0.7 mg/mL (SI: 22.8) [82]. This EO contains eucalyptol, α-pinene, limonene, spathulenol, and α-eudesmol. In our study, these compounds showed a high affinity for M^pro^, ACE2, and CatL, with a high similarity to reference drugs such as hydroxychloroquine, arbidol, and remdesivir. However, eucalyptol did not show expressive BE and DS values for any evaluated target proteins. On the other hand, the EO of *Xylopia aethiopica* A.Rich. with eucalyptol in its composition exhibited moderate antiviral action against the SARS-CoV-1 and SARS-CoV-2 pseudoviruses [57]. In another study, the EOs of *E. globulus* and *Salvia officinalis* O.Bolòs & Vigo, both containing eucalyptol as the majority compound, showed opposite effects against the H1N1 influenza virus [79].

It is noteworthy that the action of EOs is mainly attributed to their compositional complexity acting synergistically, wherein minority compounds can show higher activity than the majority ones [13,83,84]. Furthermore, the antiviral potential of EOs depends on factors concerning the virus, such as the viral load kinetic and viral protein structure. However, the whole mechanism is not fully understood yet, and it is known that the most common action mechanism is the direct interaction of the EO with the virus [13,85]. Therefore, a better understanding of the promising effects of EOs against SARS-CoV-2 is needed to aid future studies in evaluating possible action pathways of EOs against the virus.

### 3.3. Physicochemical, Pharmacokinetic, and Toxicity Properties

The structural similarity of molecules with known drugs is a widely used approach to discover promising compounds [86]. In our study, drug-likeness and bioactivity scores were used to predict the pharmacological potential of the previously selected compounds. Then, the promising compounds in both parameters were submitted to ADME/T prediction to understand the pharmacokinetics and toxicity parameters.

**Figure 3:**
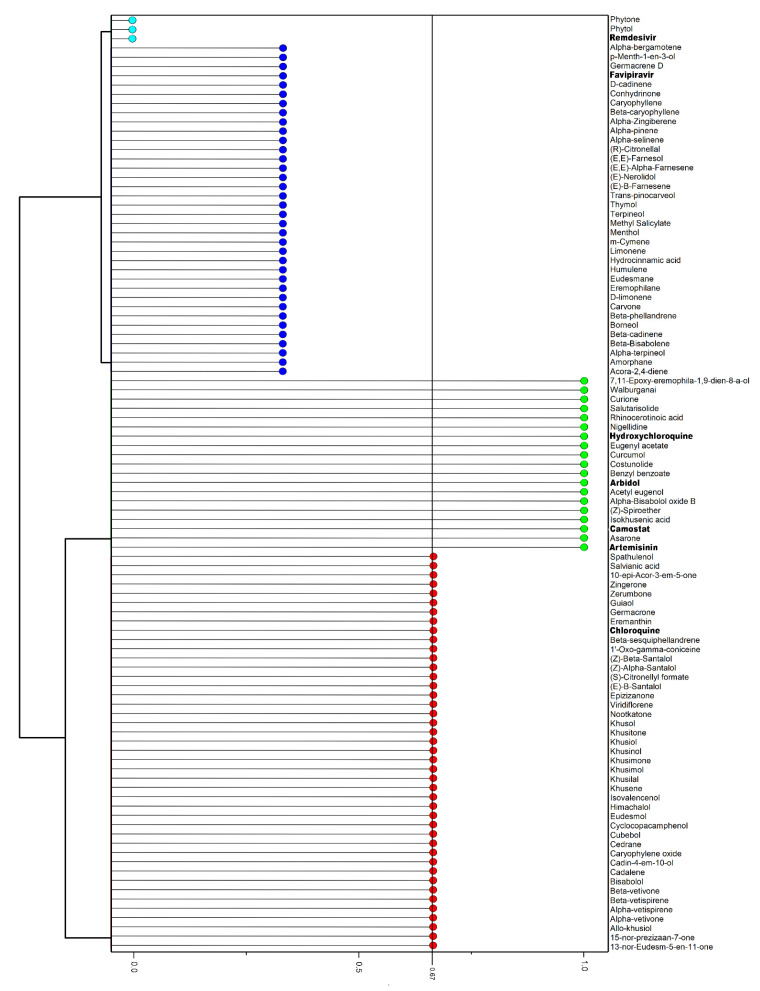
Hierarchical clustering dendrogram of drug likeness index (DLI) of essential oil (EO) compounds and reference drugs (bold) calculated from Lipinski’s, Ghose’s, and Muegge’s rules through Molinspiration (https://www.molinspiration.com/; accessed on 15 August 2021) and SwissADME (http://www.swissadme.ch/; accessed on 15 August 2021) servers.

The DLI was created based on violations of the three rules (Section 2.4.1). EO compounds and reference drugs were considered promising when they presented a DLI equal to or greater than 0.67 (Figure 3), representing the presence of violation in only one of the three rules. Of the total, 62 compounds showed a DLI greater than or equal to 0.67. Among them, 15 EO compounds had a DLI of 1.0, showing similarity with four reference drugs (artemisinin, arbidol, camostat, and hydroxychloroquine). A DLI of 0.67 was achieved by 43 EO compounds and the reference drug chloroquine, where most of them presented violations within Muegge’s rule. Lipinski’s, Ghose’s, and Muegge’s rules are parameters that qualify compounds as possible drugs. For Lipinski’s rule of five (RO5), the compound must meet the following criteria: molecular weight (MW) ≤ 500, number of hydrogen bond donors (HBD) ≤ 5, number of hydrogen bond acceptors (HBA) ≤ 10, cLogP ≤ 5, and number of rotatable bonds (n-ROTB) ≤ 10 [87]. For Ghose’s rule, the compound must have a LogP value between −0.4 and 5.6, number of atoms ranging from 20 to 70, molecular weight between 160 and 480 g/mol, and molar refractivity ranging from 40 to 130 [60]. In addition, Muegge’s rule utilizes the following criteria: MW between 200 and 600, LogP between −2 and 5, TSPA ≤ 150, number of ring ≤ 7, number of carons > 4, number of heteroatoms > 1, n-ROTB ≤ 15, HBD ≤ 5, and HBA ≤ 10 [61].

**Figure 4:**
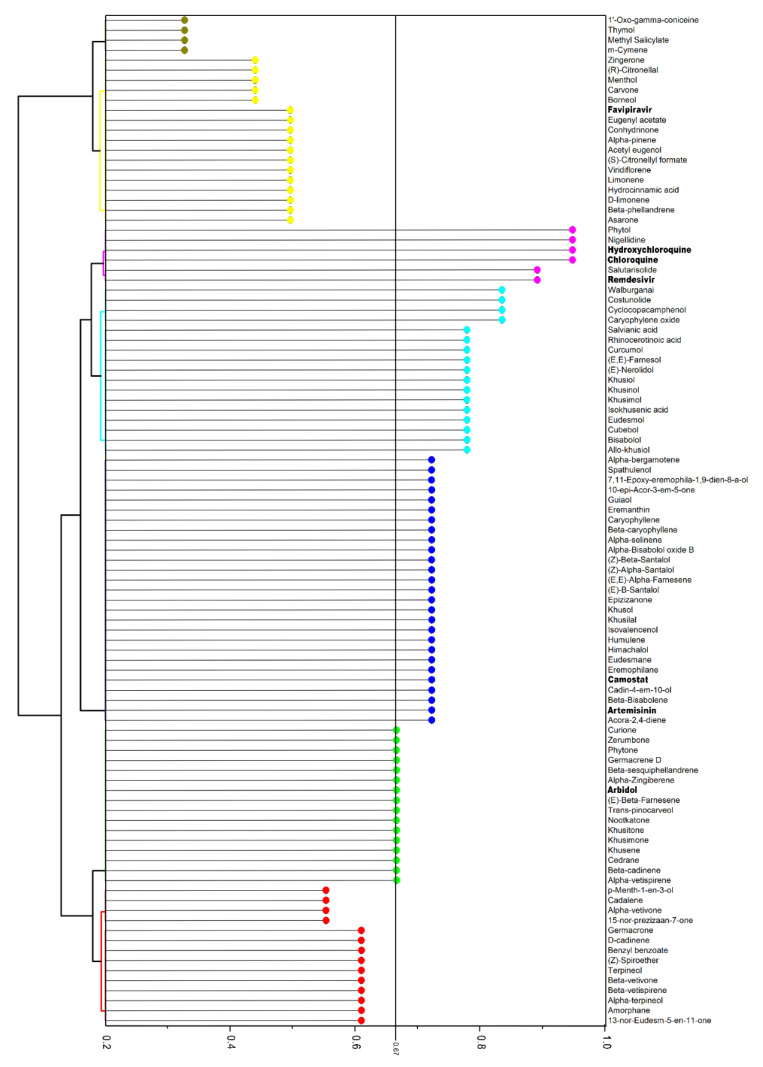
Hierarchical clustering dendrogram of bioactivity score index (BSI) of essential oil (EO) compounds and reference drugs (bold) through the Molinspiration server (https://www.molinspiration.com/; accessed on 15 August 2021).

In the same manner, the BSI was created based on the bioactivity score prediction (Section 2.4.2). The determination of bioactivity allows for the evaluation of the active potential of compounds against the main target proteins of drugs, such as binding to the G protein-coupled receptor (GPCR) ligand and nuclear receptor ligand, ion channel modulation, kinase, protease inhibition, and enzyme activity inhibition. Similar to DLI, compounds with a BSI equal to or greater than 0.67 were considered promising, referring to the active potential (Figure 4). Four clusters were formed with BSI values of 0.67 (*n* = 16), 0.72 (*n* = 27), ranging from 0.83 to 0.78 (*n* = 17), and ranging from 0.94 to 0.89 (*n* = 6). Of the total, 60 compounds showed potential activity, of which six were reference drugs (artemisinin, arbidol, camostat, remdesivir, chloroquine, and hydroxychloroquine).

After the DLI and BSI analyses, a total of 41 EO compounds and five reference drugs were selected for the next stage. Then, they were evaluated for pharmacokinetic and toxicity properties (ADME/T), which are crucial parameters for the effective selection of high-quality drug candidates (Supplementary File 3).

Concerning absorption properties, the majority of the EO compounds showed good human intestinal absorption (HIA) and Caco-2 permeability, except salvianolic acid and camostat, which showed low HIA. None of the EO compounds appeared as a substrate for P-glycoprotein (P-gp), but some acted as P-gp inhibitors (*n* = 11), including reference drugs (arbidol and camostat). According to Abdallah et al. [88], the P-gp pumps substrates out of the cells in an ATP-dependent mechanism, and the inhibition of P-gp increases the intracellular concentration of xenobiotics. Regarding distribution properties, all compounds, including reference drugs, were positive for blood–brain barrier (BBB). Only six EO compounds (costunolide, eremanthin, isokhusenic acid, rhinocerotinoic acid, salvianic acid, and walburganai) and three references drugs (arbidol, artemisinin, and camostat) showed a volume distribution (VD) out of the ideal range. However, for plasma protein binding (PPB), almost all EO compounds and reference drugs exhibited an intermediate distribution (<90%).

Regarding metabolism properties, the enzymes of cytochrome P450 of the liver and gut (CYP1A2, CYP3A4, CYP2C9, CYP2C19, and CYP2D6) are responsible for metabolizing drug molecules by breaking down, absorbing, and eliminating them through bile and urine [89]. In our study, only one EO compound (nigellidine) was inhibitory (CYP1A2), while four reference drugs demonstrated this effect as follows: arbidol (CYP1A2, CYP3A4, CYP2C9, CYP2C19, and CYP2D6), artemisinin (CYP1A2), chloroquine (CYP2D6), and hydroxychloroquine (CYP2D6). In contrast, all of the compounds were found to be substrates of at least one enzyme. The metabolization of these compounds maintains a balance of blood concentration. On the contrary, the inhibition increases the compound blood concentration, which can cause adverse effects [90].

Relating to the excretion property, all EO compounds and reference drugs had a short half-life (<3 h). Concerning toxicity properties, only one EO compound (nigellidine) was a human Ether-a-go-go Related Gene (hERG) blocker and demonstrated LD50 less than 501 mg/kg. The hERG blockers can lead to QT interval prolongation and Torsades de Pointes (TdP) arrhythmia [91].

The reference drugs hydroxychloroquine, chloroquine, arbidol, and camostat also demonstrated the ability to block hERG. Chloroquine is already known as an hERG blocker, and both chloroquine and hydroxychloroquine have been reported as potent QT interval prolongers [92]. None of the EO compounds were mutagenic (Ames). However, two reference drugs (chloroquine and hydroxychloroquine) showed mutagenic properties. For drug-induced liver injury (DILI), six EO compounds (costunolide, eremanthin, khusilal, khusitone, nigellidine, and nootkatone) and three reference drugs (arbidol, camostat, and hydroxychloroquine) demonstrated a potential to damage the liver. A drug that induces DILI can lead to acute liver failure and the need for liver transplantation [93]. According to Adegbola et al. [94], several compounds with antiviral potential were predicted to be probably mutagenic, cytotoxic, and DILI-positive. However, in our study, most EO compounds were potentially safe concerning hERG, Ames, LD50, and DILI.

**Figure 5:**
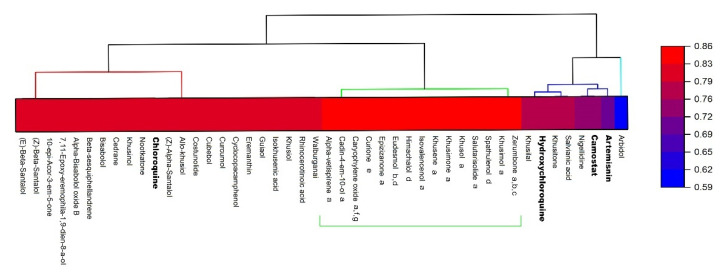
Hierarchical clustering dendrogram and heat map for the ADME/T index of essential oil (EO) compounds and reference drugs (bold) against SARS-CoV-2. Where: a Spike protein (Spro); b Angiotensin-converting enzyme-2 (ACE2); c ACE2-Spro complex; d Mpro; e Endoribonuclease (EndoU); f Nonstructural protein 9 RNA binding protein; g Replicase polyprotein.

Through HCA with the heat map graph, 15 EO compounds were grouped by their similarity, referring to the highest values of the ADME/T index, representing compounds with positive/benefit indices related to absorption, distribution, metabolism, excretion, and toxicity parameters (Figure 5). It is noteworthy that no reference drug showed this potential. Accordingly, these compounds were considered promising drug candidates for further evaluation of their anti-SARS-CoV-2 action through in vitro and in vivo studies. Likewise, such compounds may be indicators of EOs with potential antiviral activity against SARS-CoV-2.

### 3.4. Main Target Proteins for SARS-CoV-2 Inactivation

Most of the compounds with potential anti-SARS-CoV-2 action had the spike protein (S^pro^) (*n* = 11) as their main target (Figure 5). This protein is abundant on the SARS-CoV-2 surface and is responsible for mediating the entry of the virus into the human body through its binding to the human cell receptor ACE2 [95]. Compounds such as eudesmol and zerumbone have been shown to have a great affinity for ACE2. Several studies have evaluated both the S^pro^ and ACE2 as potential alternatives to inhibit viral infection. In the study by Sharbidre et al. [40], zerumbone demonstrated a great affinity for the SARS-CoV-2 spike protein (S^pro^) and human cell receptor ACE2 complex.

The main protease (M^pro^/3CL^pro^) is a cysteine protease that plays a critical role in the viral replication cycle [35,96]. Moreover, this target protein is already used to identify potential anticoronavirus inhibitors [97,98]. Eudesmol, himachalol, and spathulenol were considered promising EO compounds regarding this protein (Figure 5). Caryophyllene oxide showed a high affinity for the S^pro^, but also for RP1a and nonstructural protein 9 RNA binding protein. Moreover, curione was promising for endoribonuclease (EndoU). This protein limits the host’s immune response and is necessary for effective SARS-CoV-2 replication [99].

### 3.5. Botanical Sources of EOs with Potential Anti-SARS-CoV-2 Activity

EOs and their compounds have attracted particular attention for their reported beneficial properties such as antimicrobial, antioxidant, anti-inflammatory, antifungal, and antiviral properties [100]. Their antiviral properties have been increasingly researched due to the current COVID-19 pandemic scenario worldwide. According to Queiroz de Oliveira et al. [101], the pharmaceutical and food industries are highly interested in finding EOs with potential anti-SARS-CoV-2 activity. However, in vitro and in vivo studies about this subject are scarce in the literature. In this context, our findings are helpful to identify promising botanical sources for obtaining effective EOs against SARS-CoV-2 and other viral infections.

Eudesmol is present in large amounts in EOs extracted from plants such as *Neocallitropsis pancheri* Laub., *Atractylodes lancea* Kitam., and *Melaleuca leucadendra* Cheel [102,103]. The antiviral action of this compound was observed against herpes simplex virus type 1 (HSV-1) with an IC50 of 6 µg/mL and an antiviral selectivity index (SI) of 5.8 [83]. In the study by Senthil Kumar et al. [56], eudesmol was present in the EO from *P. graveolens* and showed significant inhibitory effects of ACE2 on epithelial cells. This compound has been suggested for incorporation into biodegradable food packaging to mitigate viral cross-contamination [101].

Caryophyllene oxide is reported in the EO from the genera *Cinnamomum verum* J.Presl, *Syzygium aromaticum* Merr. & L.M.Perry, *M. officinalis*, *Zataria multiflora* Boiss., and *Eucaliptos* [104,105,106]. The EO from *Z. multiflora*, with caryophyllene oxide in its composition, showed satisfactory antiviral action against HSV-1 [81], which was also observed for spathulenol, a majority compound in *Eucalyptus polybractea* F.Muell. ex R.T.Baker, *Baccharis dracunculifolia* DC., and varieties of *Eugenia brasiliensis* Cambess [81,103,107].

The species *Zingiber zerumbet* Triboun & K.Larsen, together with those belonging to the genus *Curcuma*, are known for their vast bioactive potential, containing compounds such as curione and zerumbone [108,109,110]. The antiviral action of EOs obtained from plants from the *Curcuma* genus is little reported in the literature. Maurya et al. [111] reported the effectiveness of EO from *Curcuma longa* Velay., Pandrav., J.K.George & Varapr. against papaya ringspot virus (PRSV). The same potential was found for zerumbone against the Epstein–Barr Virus (EBV) [112].

The EO of *Cedrus libani* A.Rich, rich in himachalol, showed action against HSV-1 [113]. Cadin-4-en-10-ol (alpha-Cadinol) is predominant in plants from the *Eugenia* genus such as *E. biflora* DC. and *E. brasiliensis* [107]. Salutarisolide was found in large amounts in the EO from *Warburgia salutaris* (G.Bertol.) Chiov. [114]. However, there are no reports about the antiviral effects of either cadin-4-en-10-ol (alpha-cadinol) nor salutarisolide at the present moment.

Most of the promising compounds found in the present study were significantly identified in vetiver EO (*Chrysopogon zizanioides* Roberty or *Vetiveria zizanoides* Nash) as alpha-vetispirene, isovalencenol, khusene, khusimol, khusimone, khusol, and epizizanone [103,115,116,117]. This EO has been reported as antimicrobial [118], antifungal [119], anxiolytic [120], antioxidant, and anti-inflammatory [121]. To our knowledge, there is only one study reporting the antiviral action of this EO. Ralambondrainy et al. [85] observed no antiviral activity in vitro of *V. zizanoides* at 29.4 μg/mL against the Ross River virus (RRV). Nevertheless, the maximum non-cytotoxic concentration of the EO from *V. zizanoides* was not determined, and thus, it was not tested, which may have contributed to their non-significant antiviral action results.

The antiviral action of EOs strongly depends on their composition, concentration, and mechanism of action against the virus [13], as well as aspects regarding the virus such as the viral load kinetic and viral protein structure. Despite this fact, no study has evaluated the antiviral action of the promising EO compounds identified in the present study against SARS-CoV-2, indicating a clear need for future studies in this subject.

## 4. Review Limitations

This review aimed to conduct a survey of the literature on the potential of EO compounds against the SARS-CoV-2 virus; however, it presents some limitations. As expected, because the COVID-19 pandemic is recent, most of the data retrieved were from in silico studies against the main target proteins of the virus. In silico data have great value in the virtual screening of new drugs. However, they can be obtained by different methods [122], so data from in silico studies must be validated through in vitro and in vivo approaches, which are imperative for assessing the antiviral potential of EOs against the SARS-CoV-2 virus. Furthermore, our study was not registered in PROSPERO. This is due to the unavoidable need to find new, effective compounds against SARS-CoV-2 and the fact that recent reports related to COVID-19 are being published daily. Despite the matters mentioned above, this study can be updated anytime by incorporating new data and relevant information that can help to better understand the potential of EOs against SARS-CoV-2, presenting an adequate record of the research.

## 5. Conclusions and Future Perspectives

Ninety-four EO compounds showed a high binding affinity against 11 target proteins. Amongst them, 15 (alpha-vetispirene, cadin-4-em-10-ol, caryophyllene oxide, eudesmol, himachalol, isovalencenol, khusene, khusimol, khusimone, khusol, epizizanone, salutarisolide, zerumbone, curione, and spathulenol) were considered the most promising EO compounds against SARS-CoV-2 based on their physicochemical, pharmacokinetic, and toxicity properties. Considering these findings, several plants were suggested as EO sources with potential anti-SARS-CoV-2, such as *M. officinalis, Z. multiflora, E. brasiliensis, Z. zerumbet, C. libani*, and *V. zizanoides*. The EO from *V. zizanoides* showed the highest number of compounds with anti-SARS-CoV-2 potential and the S^pro^ as their target protein. It is noteworthy that the data regarding the action of EOs and their isolated compounds against the COVID-19 virus are still recent and preliminary. Therefore, further in vitro and in vivo studies are needed to determine the antiviral activity of the compounds and elucidate their possible mechanisms of action. Our findings are helpful to aid further in vitro and in vivo studies to identify effective EOs and guide the development of novel formulations against COVID-19 for different food, pharmaceutical, cosmetic, and medical applications.

## Data Availability

Data sharing not applicable.

## Supplementary Materials

The following are available online at https://www.mdpi.com/article/10.3390/ph14111138/s1, Supplementary File 1: PRISMA 2020 Checklist; Supplementary File 2: Pharmacokinetic and toxicology properties of essential oil (EO) compounds and reference drugs obtained through the ADMETlab server; Supplementary File 3: Hierarchical clustering dendrogram and heat map for essential oil compounds and reference drugs against the main target proteins of SARS-CoV-2.
