# Supplementary material for: An Evaluation of the Potential of Essential Oils against SARS-CoV-2 from In Silico Studies through the Systematic Review Using a Chemometric Approach
Source: Pharmaceuticals (Basel). 2021 Nov 10;14(11):1138. doi: 10.3390/ph14111138 (PMC8624289; doi:10.3390/ph14111138)
Supplement: Supplementary file 1 [file pharmaceuticals-14-01138-s001.zip › Supplementary file 2.pdf]

Supplementary file 2

Table S1 Pharmacokinetic and toxicology properties of essential oil (EO) compounds and reference drugs obtained through the ADMETlab server (http://admet.scbdd.com/).

	Absorption				Distribution			Metabolism										Excretion	Toxicity			
**EO compounds**	**Caco-2 permeability**	**Pgp-inhibitor***	**Pgp-substrate***	**HIA***	**PPB**	**BBB***	**VD**	**CYP450 1A2 inhibitor***	**CYP450 1A2 substrate***	**CYP450 3A4 inhibitor***	**CYP450 3A4 substrate***	**CYP450 2C9 inhibitor***	**CYP450 2C9 substrate***	**CYP450 2C19 inhibitor***	**CYP450 2C19 substrate***	**CYP450 2D6 inhibitor***	**CYP450 2D6 substrate***	**T 1/2**	**hERG***	**Ames***	**LD50**	**DILI ***
(E)-B-Santalol	-4.40	0.57	0.05	0.86	85.6	0.95	0.29	0.02	0.39	0.07	0.50	0.08	0.39	0.23	0.56	0.31	0.54	1.67	0.38	0.20	1914.8	0.21
(Z)-Alpha-Santalol	-4.45	0.73	0.07	0.07	83.5	0.88	0.52	0.03	0.42	0.05	0.51	0.16	0.28	0.13	0.53	0.32	0.45	1.84	0.38	0.29	1180.1	0.18
(Z)-Beta-Santalol	-4.40	0.57	0.05	0.86	85.6	0.95	0.29	0.02	0.39	0.07	0.50	0.08	0.39	0.23	0.56	0.31	0.54	1.67	0.38	0.20	1914.8	0.21
10-epi-Acor-3-em-5-one	-4.38	0.56	0.04	0.85	80.4	0.95	0.37	0.13	0.39	0.03	0.61	0.03	0.40	0.18	0.50	0.25	0.27	1.64	0.30	0.02	1552.9	0.45
7,11-Epoxy-eremophila-1,9-dien-8-a-ol	-4.41	0.19	0.12	0.81	71.8	0.95	0.39	0.10	0.32	0.05	0.46	0.07	0.34	0.22	0.54	0.27	0.39	1.68	0.42	0.44	924.4	0.36
Allo-khusiol	-4.42	0.12	0.08	0.91	75.8	0.96	0.55	0.02	0.24	0.02	0.40	0.02	0.28	0.10	0.38	0.23	0.32	1.60	0.32	0.08	1738.1	0.13
Alpha-Bisabolol oxide B	-4.45	0.51	0.02	0.81	79.4	0.98	0.39	0.11	0.43	0.08	0.65	0.08	0.42	0.22	0.53	0.40	0.46	1.57	0.38	0.18	1902.2	0.25
Alpha-vetispirene	-4.46	0.31	0.04	0.86	71.1	0.97	0.52	0.19	0.48	0.06	0.56	0.06	0.39	0.24	0.53	0.38	0.42	1.99	0.39	0.07	3476.0	0.27
Beta-sesquiphellandrene	-4.45	0.58	0.03	0.83	78.7	0.92	0.44	0.10	0.32	0.02	0.56	0.04	0.35	0.14	0.52	0.34	0.48	1.79	0.46	0.05	5500.4	0.21
Bisabolol	-4.42	0.64	0.03	0.89	86.0	0.86	0.40	0.06	0.26	0.06	0.53	0.07	0.40	0.34	0.43	0.36	0.54	1.58	0.45	0.14	3460.0	0.15
Cadin-4-em-10-ol	-4.42	0.46	0.09	0.91	80.1	0.95	0.49	0.02	0.49	0.04	0.64	0.03	0.33	0.22	0.61	0.35	0.42	1.61	0.35	0.04	1879.6	0.21
Caryophylene oxide	-4.41	0.25	0.01	0.78	78.5	0.99	0.60	0.09	0.39	0.04	0.63	0.03	0.28	0.08	0.65	0.32	0.37	1.93	0.32	0.05	1422.8	0.32
Cedrane	-4.44	0.25	0.17	0.86	70.7	0.98	0.62	0.04	0.46	0.09	0.46	0.03	0.34	0.11	0.54	0.30	0.46	1.98	0.34	0.20	2504.1	0.18
Costunolide	-4.29	0.51	0.03	0.75	76.4	0.99	-0.07	0.12	0.54	0.06	0.58	0.04	0.46	0.20	0.71	0.24	0.32	1.50	0.46	0.11	2748.4	0.51
Cubebol	-4.41	0.27	0.13	0.89	75.7	0.82	0.63	0.03	0.36	0.15	0.50	0.05	0.18	0.09	0.39	0.23	0.38	1.73	0.34	0.19	1588.8	0.16
Curcumol	-4.43	0.28	0.11	0.81	75.1	0.76	0.27	0.13	0.35	0.08	0.40	0.08	0.27	0.18	0.47	0.31	0.36	1.62	0.27	0.18	1323.0	0.31
Curione	-4.31	0.42	0.29	0.74	79.5	0.94	0.11	0.10	0.57	0.02	0.46	0.04	0.31	0.06	0.51	0.23	0.38	1.56	0.38	0.09	2379.9	0.35
Cyclocopacamphenol	-4.40	0.44	0.04	0.87	70.8	0.94	0.56	0.01	0.35	0.10	0.49	0.04	0.22	0.04	0.47	0.29	0.28	1.78	0.33	0.31	1233.5	0.18
Epizizanone	-4.36	0.18	0.04	0.86	77.6	0.97	0.38	0.02	0.44	0.07	0.59	0.02	0.29	0.06	0.57	0.20	0.28	1.75	0.30	0.06	1611.1	0.43
Eremanthin	-4.31	0.32	0.03	0.76	69.4	0.99	-0.06	0.16	0.49	0.07	0.55	0.03	0.41	0.13	0.65	0.28	0.37	1.34	0.29	0.15	800.4	0.51

Eudesmol	-4.44	0.21	0.01	0.90	78.6	0.98	0.38	0.03	0.40	0.12	0.64	0.07	0.35	0.22	0.52	0.32	0.33	1.62	0.34	0.06	1754.2	0.22
Guiaol	-4.45	0.44	0.02	0.84	80.5	0.96	0.42	0.08	0.36	0.04	0.46	0.08	0.21	0.30	0.45	0.34	0.43	1.61	0.32	0.15	2018.7	0.23
Himachalol	-4.44	0.37	0.01	0.88	80.9	0.99	0.49	0.10	0.45	0.07	0.71	0.01	0.34	0.25	0.59	0.38	0.40	1.62	0.34	0.13	1412.8	0.20
Isokhusenic acid	-4.56	0.18	0.01	0.85	80.3	0.85	-0.27	0.01	0.30	0.02	0.59	0.02	0.28	0.07	0.48	0.22	0.33	1.56	0.30	0.24	1438.3	0.45
Isovalencenol	-4.43	0.28	0.12	0.88	84.1	0.94	0.32	0.03	0.52	0.09	0.58	0.06	0.36	0.05	0.50	0.38	0.34	1.70	0.38	0.23	1923.7	0.19
Khusene	-4.44	0.21	0.06	0.86	74.3	0.97	0.58	0.02	0.41	0.08	0.51	0.04	0.30	0.07	0.55	0.33	0.44	2.00	0.33	0.14	2769.5	0.24
Khusilal	-4.26	0.52	0.01	0.84	75.3	0.97	0.28	0.10	0.45	0.13	0.44	0.14	0.45	0.27	0.48	0.30	0.39	1.59	0.25	0.32	2679.1	0.55
Khusimol	-4.39	0.19	0.03	0.86	78.0	0.95	0.37	0.01	0.39	0.03	0.57	0.02	0.35	0.06	0.50	0.30	0.52	1.61	0.33	0.19	1517.5	0.20
Khusimone	-4.34	0.25	0.01	0.86	76.3	0.97	0.35	0.04	0.45	0.09	0.59	0.05	0.32	0.08	0.65	0.19	0.30	1.76	0.29	0.06	1600.6	0.48
Khusinol	-4.41	0.29	0.14	0.87	83.6	0.89	0.37	0.03	0.33	0.05	0.52	0.05	0.36	0.15	0.43	0.36	0.40	1.50	0.36	0.13	1710.5	0.24
Khusiol	-4.42	0.12	0.08	0.91	75.8	0.96	0.55	0.02	0.24	0.02	0.40	0.02	0.28	0.10	0.38	0.23	0.32	1.60	0.32	0.08	1738.1	0.13
Khusitone	-4.34	0.43	0.00	0.87	79.7	0.98	0.29	0.09	0.42	0.04	0.45	0.03	0.45	0.40	0.46	0.29	0.35	1.55	0.26	0.22	2450.7	0.52
Khusol	-4.39	0.42	0.06	0.86	82.3	0.85	0.27	0.04	0.48	0.09	0.53	0.03	0.44	0.11	0.57	0.37	0.44	1.48	0.36	0.28	1928.1	0.28
Nigellidine	-4.69	0.37	0.02	0.78	85.4	0.81	0.73	0.64	0.40	0.26	0.47	0.41	0.52	0.37	0.47	0.29	0.48	1.77	0.71	0.44	417.7	0.54
Nootkatone	-4.38	0.79	0.02	0.89	80.8	0.97	0.37	0.07	0.52	0.02	0.70	0.03	0.25	0.16	0.67	0.17	0.22	1.61	0.32	0.03	1950.4	0.56
Rhinocerotinoic acid	-4.68	0.83	0.05	0.80	94.1	0.90	-0.37	0.03	0.32	0.10	0.53	0.09	0.31	0.20	0.38	0.27	0.29	1.75	0.48	0.07	580.8	0.23
Salutarisolide	-4.46	0.43	0.04	0.75	65.4	0.94	0.08	0.04	0.42	0.11	0.68	0.05	0.42	0.19	0.60	0.27	0.20	1.37	0.26	0.20	567.6	0.49
Salvianic acid	-5.89	0.03	0.19	0.48	30.1	0.77	-0.67	0.01	0.17	0.02	0.12	0.03	0.59	0.02	0.34	0.24	0.62	0.60	0.14	0.45	2157.9	0.16
Spathulenol	-4.42	0.18	0.01	0.83	79.3	0.98	0.53	0.06	0.43	0.08	0.66	0.02	0.40	0.19	0.54	0.27	0.37	1.57	0.32	0.16	1199.9	0.17
Walburganai	-4.47	0.25	0.01	0.75	68.9	0.98	-0.13	0.07	0.43	0.31	0.57	0.06	0.31	0.33	0.49	0.36	0.35	1.60	0.23	0.13	1108.0	0.31
Zerumbone	-4.33	0.25	0.05	0.79	78.8	0.99	0.46	0.16	0.64	0.06	0.59	0.08	0.50	0.38	0.68	0.29	0.26	1.75	0.41	0.08	2472.5	0.32
**Reference drugs**																						
Arbidol	-4.89	0.81	0.24	0.70	91.6	0.59	-0.08	0.64	0.45	0.67	0.64	0.80	0.37	0.87	0.55	0.56	0.43	1.77	0.89	0.22	629.4	0.59
Artemisinin	-4.50	0.35	0.04	0.64	61.5	0.93	-0.18	0.97	0.43	0.07	0.64	0.05	0.08	0.06	0.64	0.20	0.60	1.23	0.27	0.33	3025.3	0.39
Camostat	-5.39	0.57	0.17	0.39	78.8	0.67	-0.85	0.08	0.60	0.06	0.62	0.17	0.40	0.06	0.54	0.42	0.50	1.27	0.51	0.25	1867.8	0.62
Chloroquine	-4.57	0.26	0.81	0.88	85.4	0.95	1.26	0.03	0.74	0.07	0.83	0.05	0.08	0.19	0.71	0.66	0.85	2.23	0.88	0.81	729.4	0.47
Hydroxychloroquine	-4.75	0.28	0.80	0.81	84.1	0.85	1.07	0.05	0.68	0.12	0.33	0.10	0.06	0.09	0.64	0.63	0.32	2.08	0.92	0.72	787.4	0.50

Interpretation provided by ADMETlab server: Caco-2 permeability (Green: higher than -5.15 Log); P-glycoprotein (P-gp) (Green: non-inhibidor, Red: inihbidor; Green: substrate, Red: non-substrate); Human Intestinal Absorption (HIA) (Green: HIA+, Red: HIA-); Plasma Protein Binding (PPB) (Green: > 90%, Red: < 90%); Blood–Brain Barrier (BBB) (Green: BBB+, Red: BBB-); Volume Distribution (VD) (Green: between 0.04-20 L kg^{-1}); CYP450 (Green: non-Inhibidor, Red: inhibidor); Half-life (T ½) (Green: < 3 hours); hERG blockers (Green: low-blockers, Red: blockers); Ames mutagenicity (Green: ames-, Red: ames+); LD50 (Green: > 501 mg Kg^{-1}, Red: < 501 mg Kg^{-1}); Drug Induced Liver Injury (DILI) (Green: DILI-, Red: DILI+).* Expected probability for categorical variables.
