# Supplementary material for: An Evaluation of the Potential of Essential Oils against SARS-CoV-2 from In Silico Studies through the Systematic Review Using a Chemometric Approach
Source: Pharmaceuticals (Basel). 2021 Nov 10;14(11):1138. doi: 10.3390/ph14111138 (PMC8624289; doi:10.3390/ph14111138)
Supplement: Supplementary file 1 [file pharmaceuticals-14-01138-s001.zip › Supplementary file 3.pptx]

## Slide 1
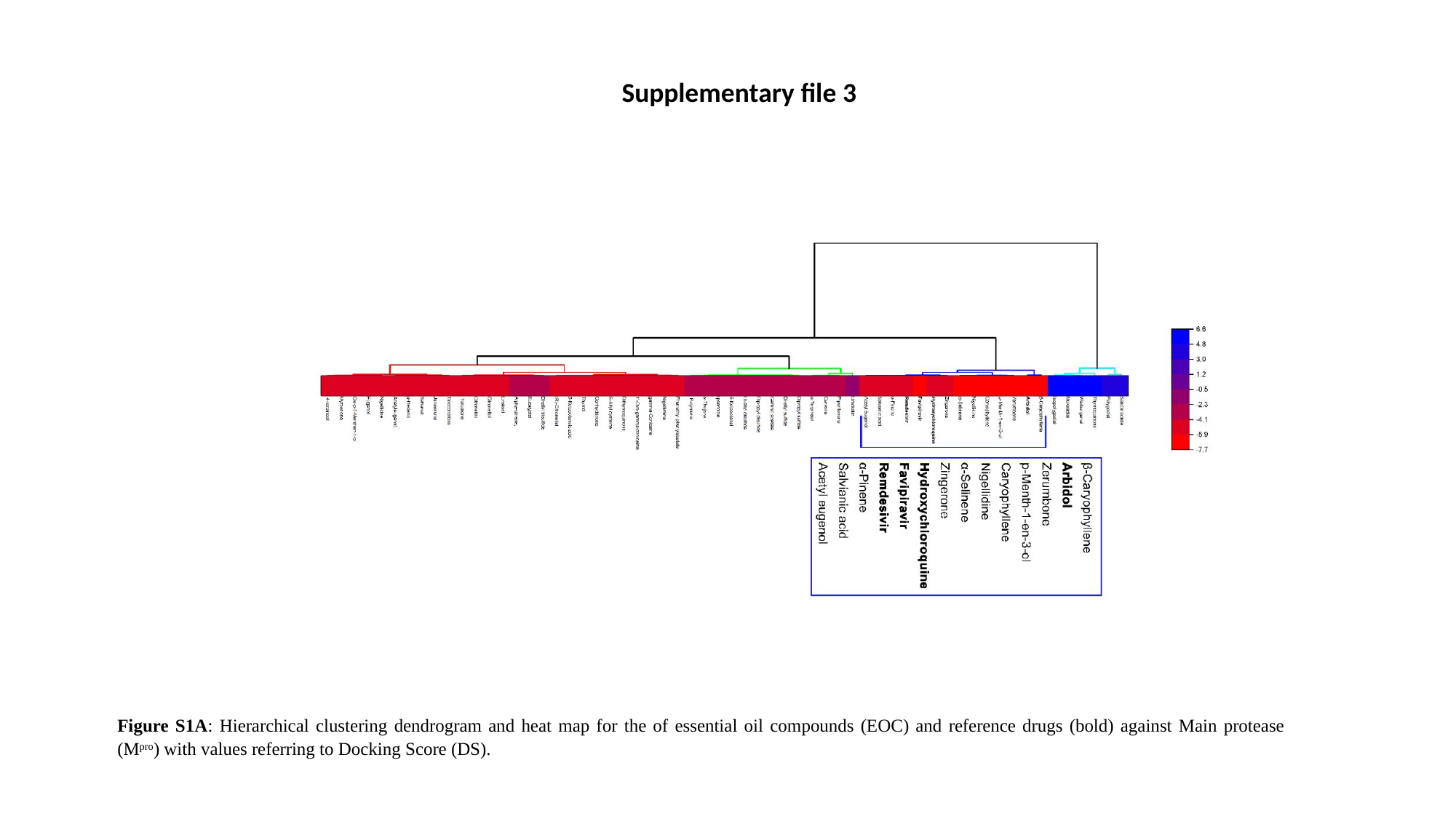

Supplementary file 3
Figure S1A: Hierarchical clustering dendrogram and heat map for the of essential oil compounds (EOC) and reference drugs (bold) against Main protease (Mpro) with values referring to Docking Score (DS).

## Slide 2
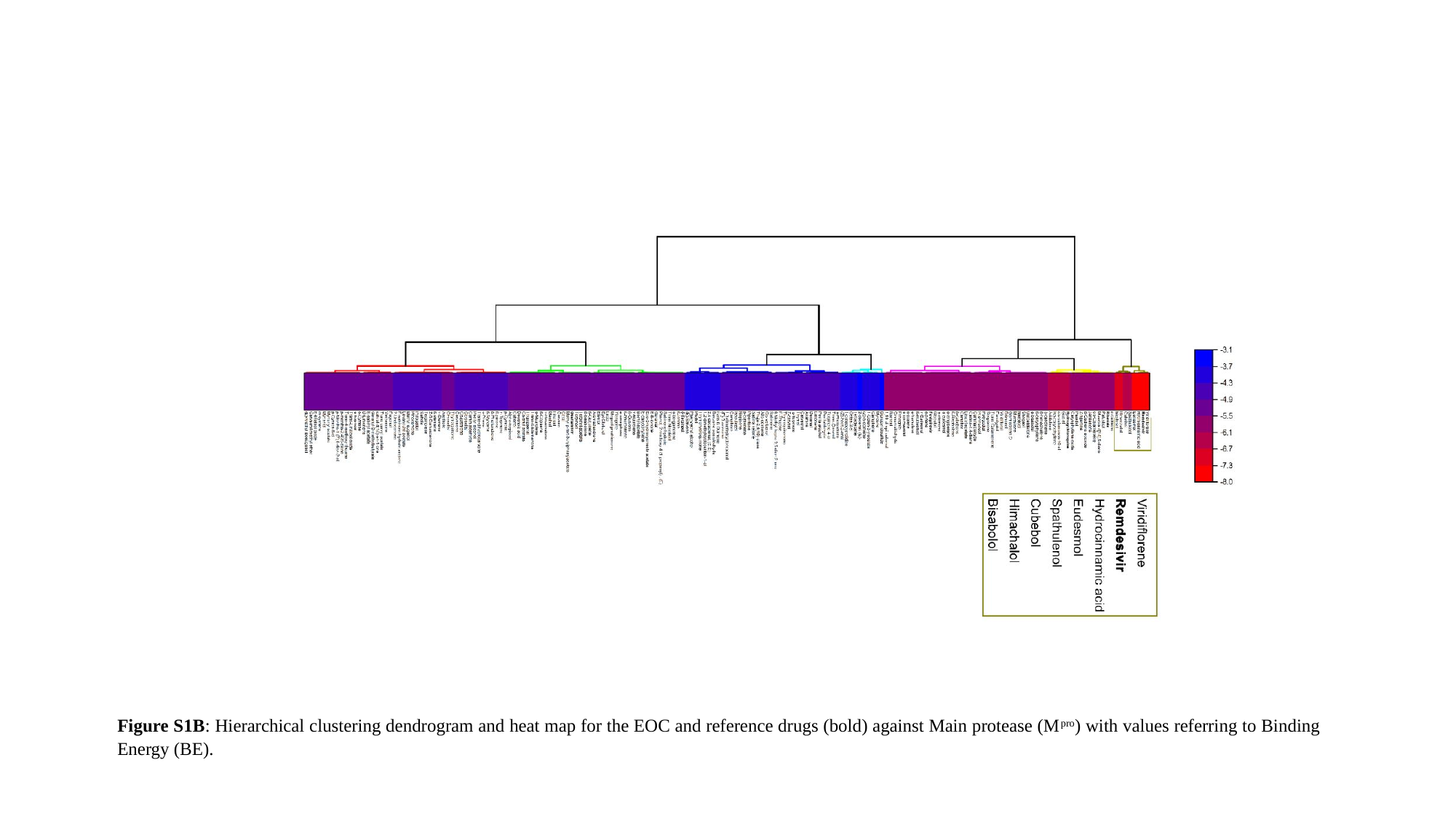

Figure S1B: Hierarchical clustering dendrogram and heat map for the EOC and reference drugs (bold) against Main protease (Mpro) with values referring to Binding Energy (BE).

## Slide 3
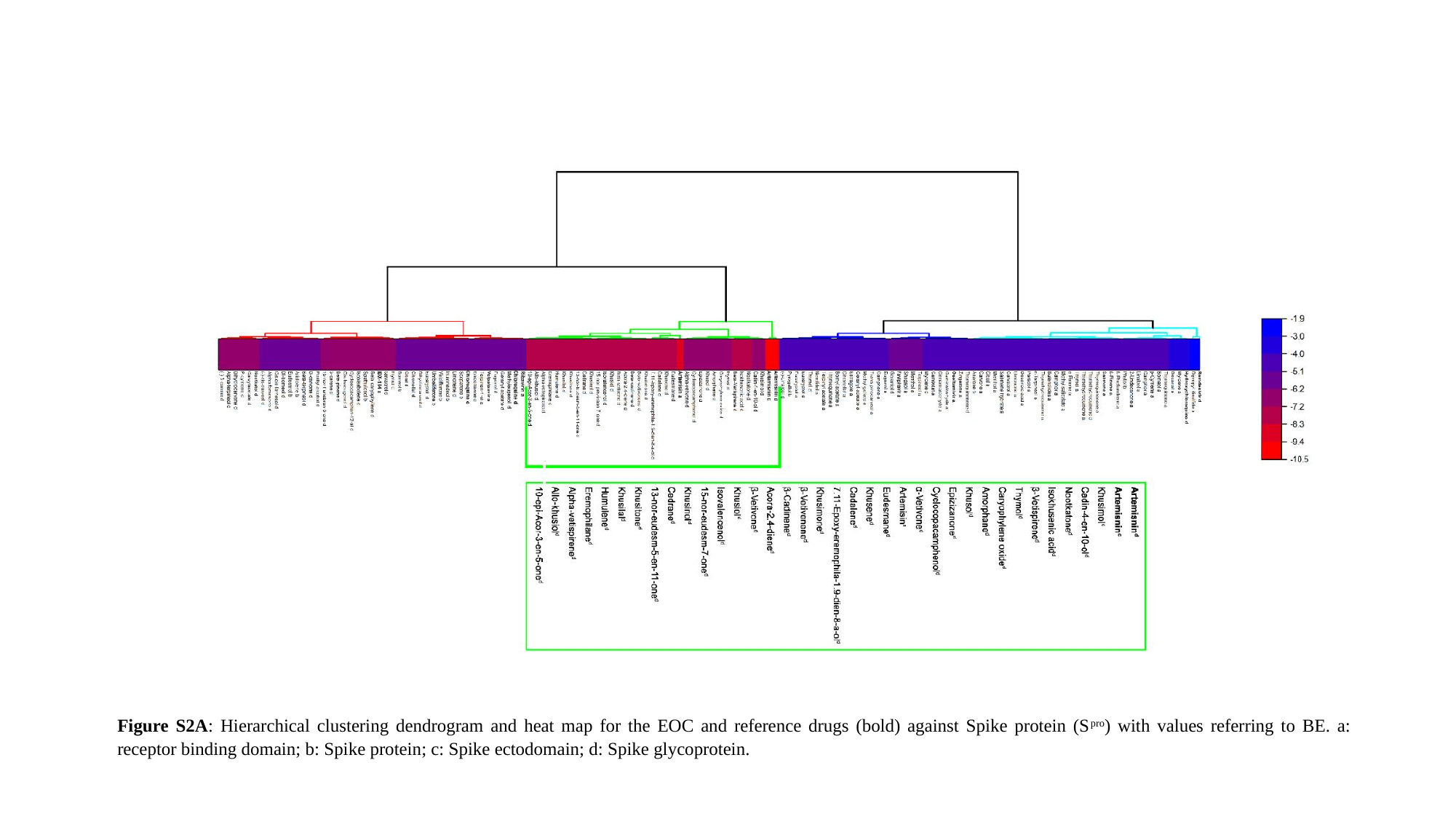

Figure S2A: Hierarchical clustering dendrogram and heat map for the EOC and reference drugs (bold) against Spike protein (Spro) with values referring to BE. a: receptor binding domain; b: Spike protein; c: Spike ectodomain; d: Spike glycoprotein.

## Slide 4
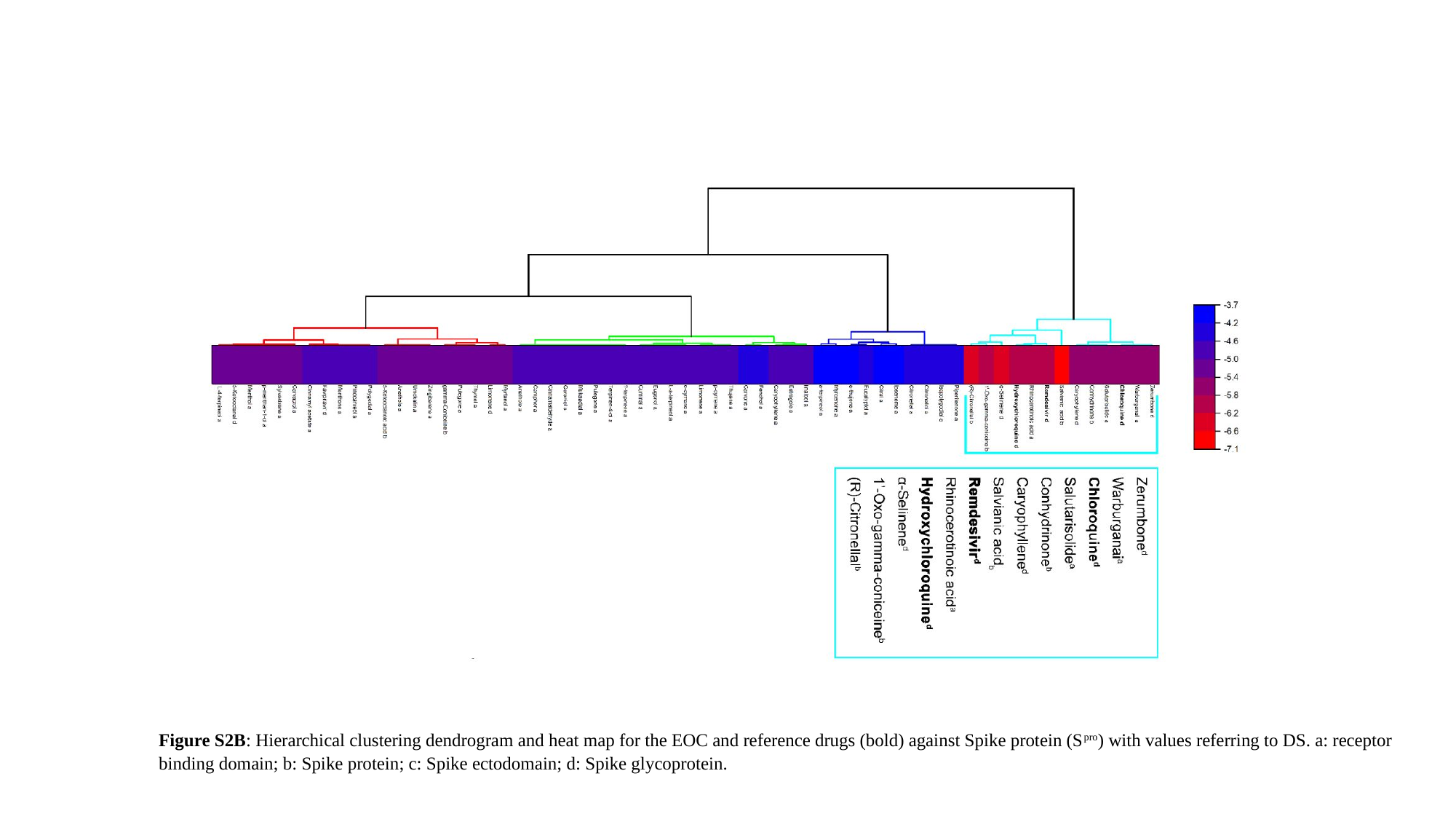

Figure S2B: Hierarchical clustering dendrogram and heat map for the EOC and reference drugs (bold) against Spike protein (Spro) with values referring to DS. a: receptor binding domain; b: Spike protein; c: Spike ectodomain; d: Spike glycoprotein.

## Slide 5
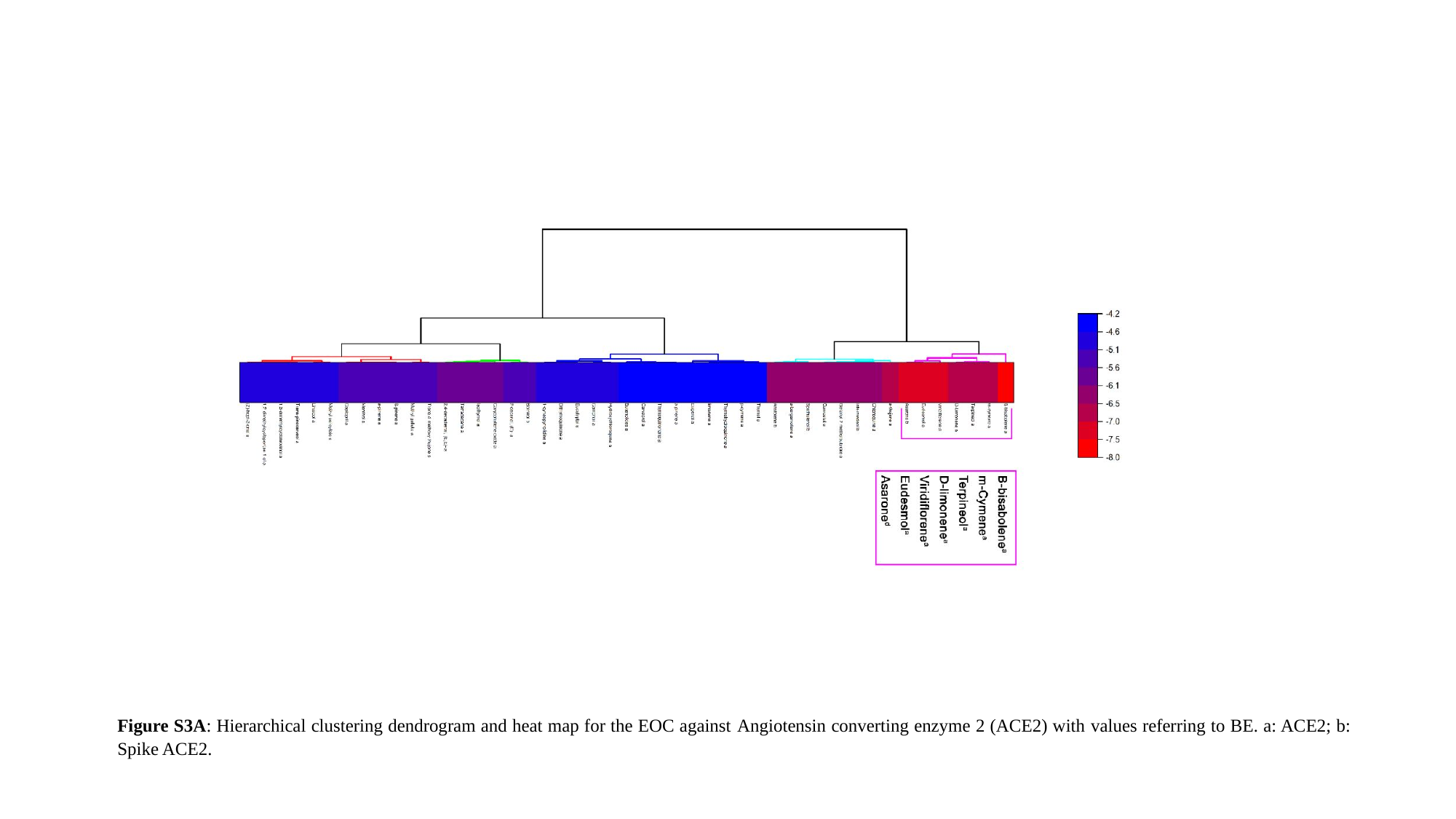

Figure S3A: Hierarchical clustering dendrogram and heat map for the EOC against Angiotensin converting enzyme 2 (ACE2) with values referring to BE. a: ACE2; b: Spike ACE2.

## Slide 6
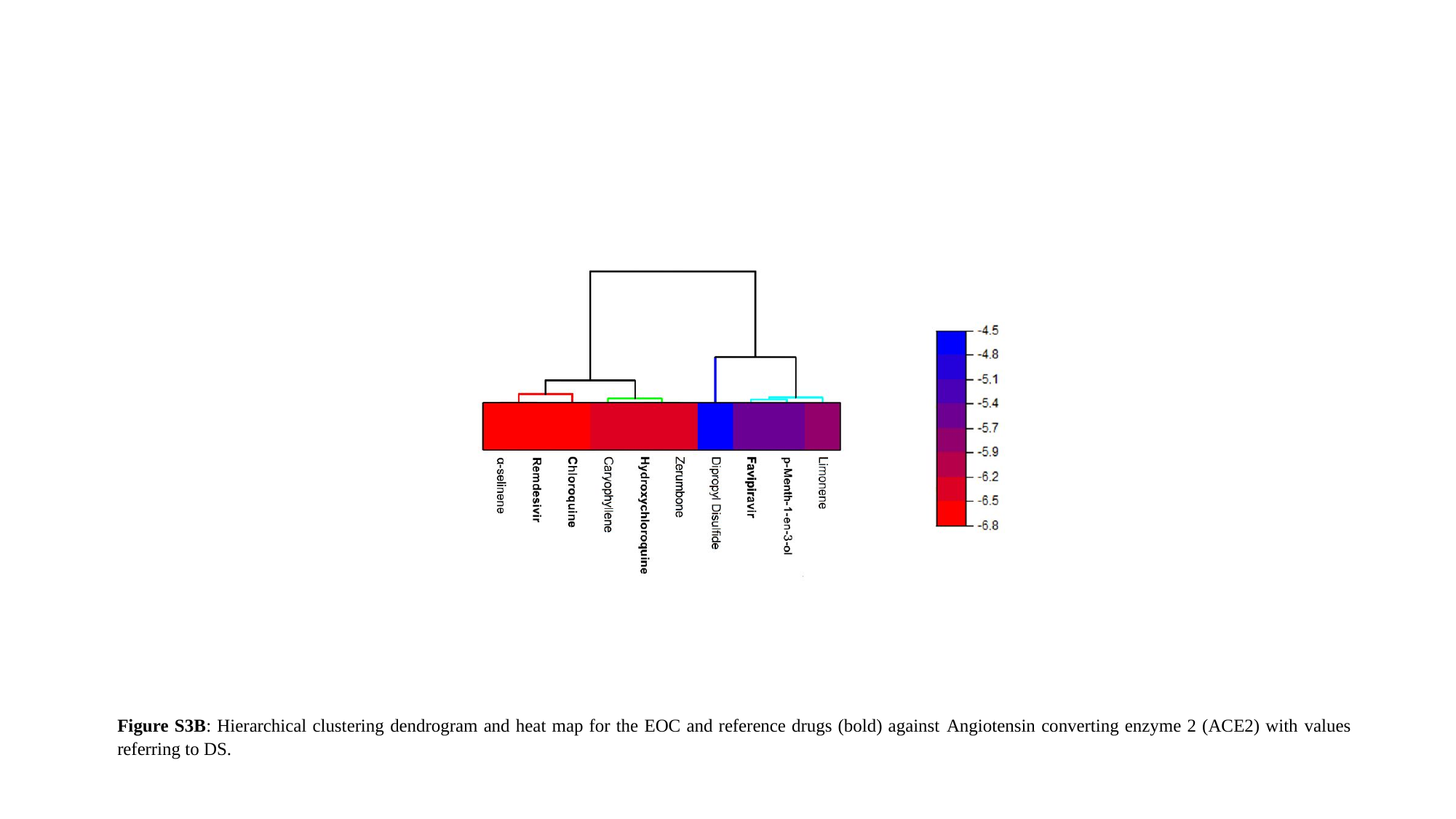

Figure S3B: Hierarchical clustering dendrogram and heat map for the EOC and reference drugs (bold) against Angiotensin converting enzyme 2 (ACE2) with values referring to DS.

## Slide 7
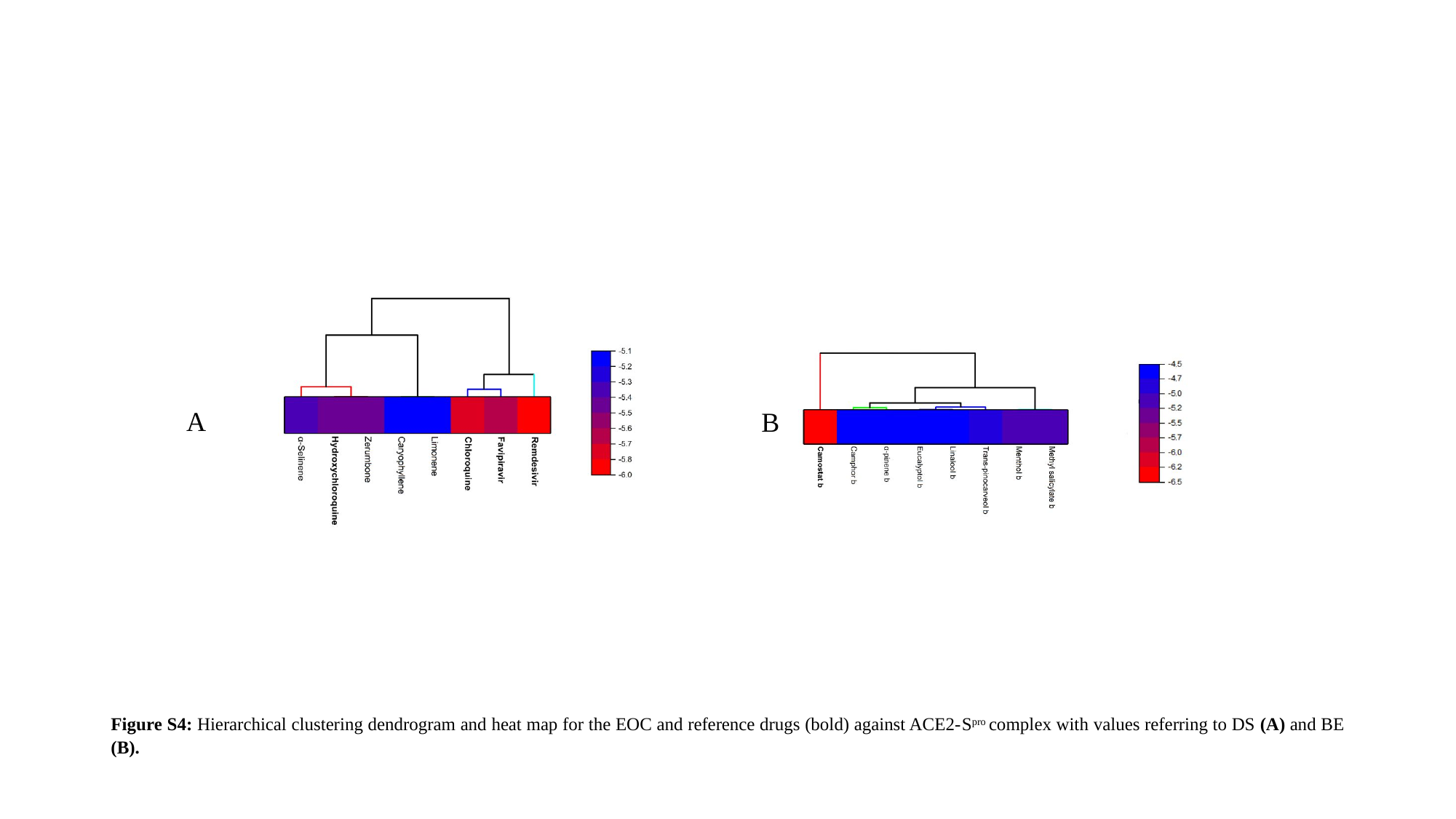

A
B
Figure S4: Hierarchical clustering dendrogram and heat map for the EOC and reference drugs (bold) against ACE2-Spro complex with values referring to DS (A) and BE (B).

## Slide 8
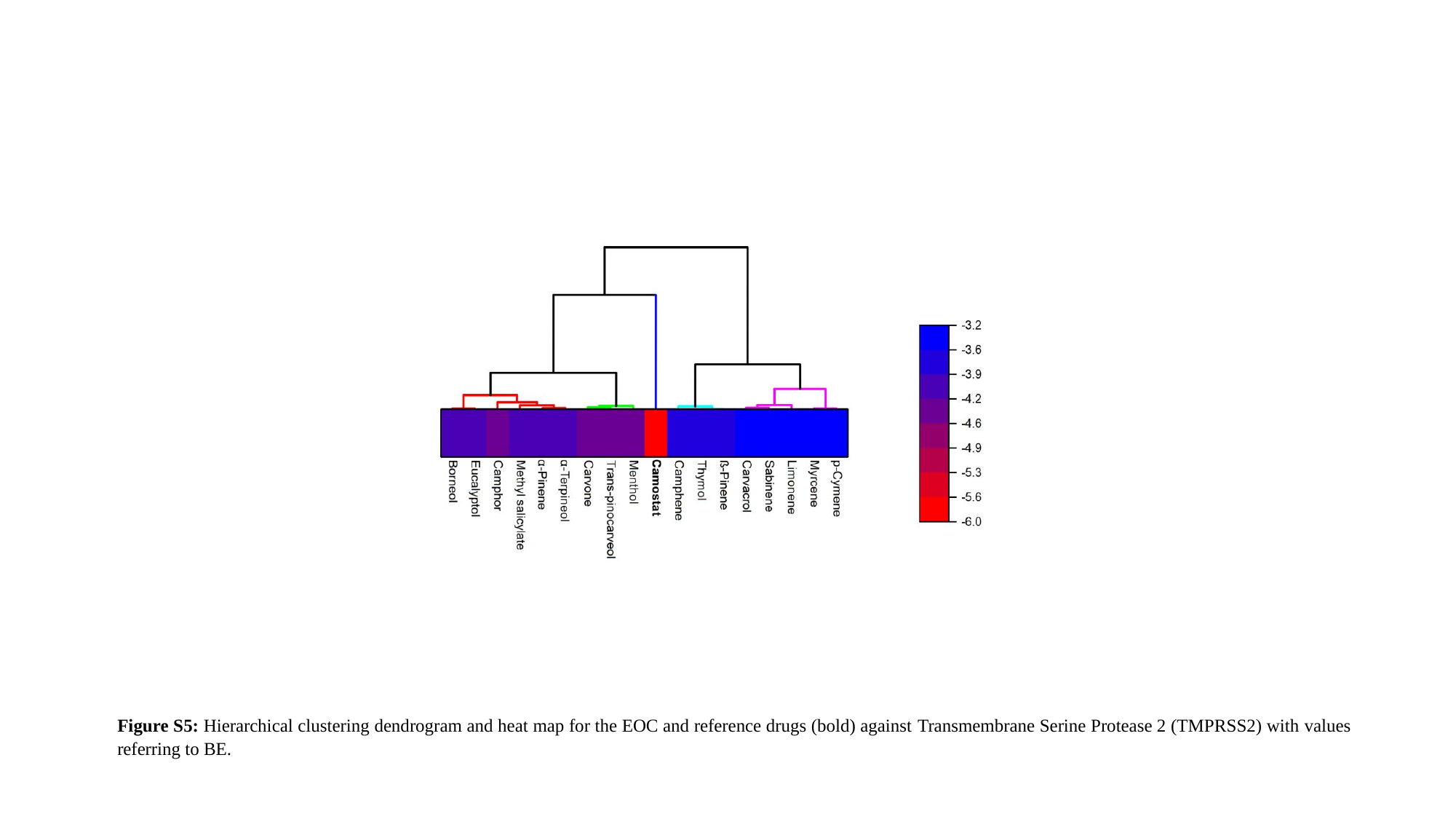

Figure S5: Hierarchical clustering dendrogram and heat map for the EOC and reference drugs (bold) against Transmembrane Serine Protease 2 (TMPRSS2) with values referring to BE.

## Slide 9
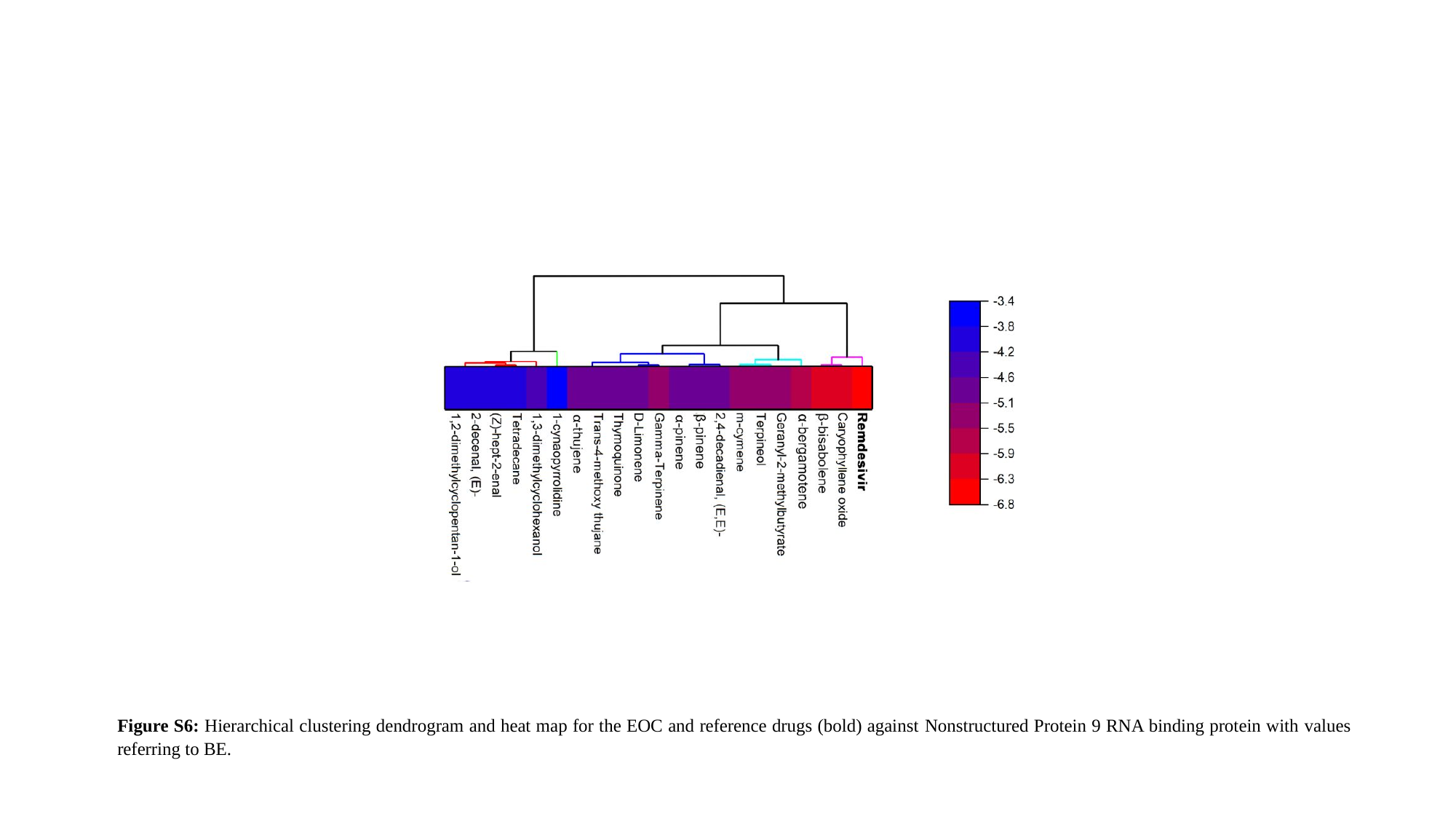

Figure S6: Hierarchical clustering dendrogram and heat map for the EOC and reference drugs (bold) against Nonstructured Protein 9 RNA binding protein with values referring to BE.

## Slide 10
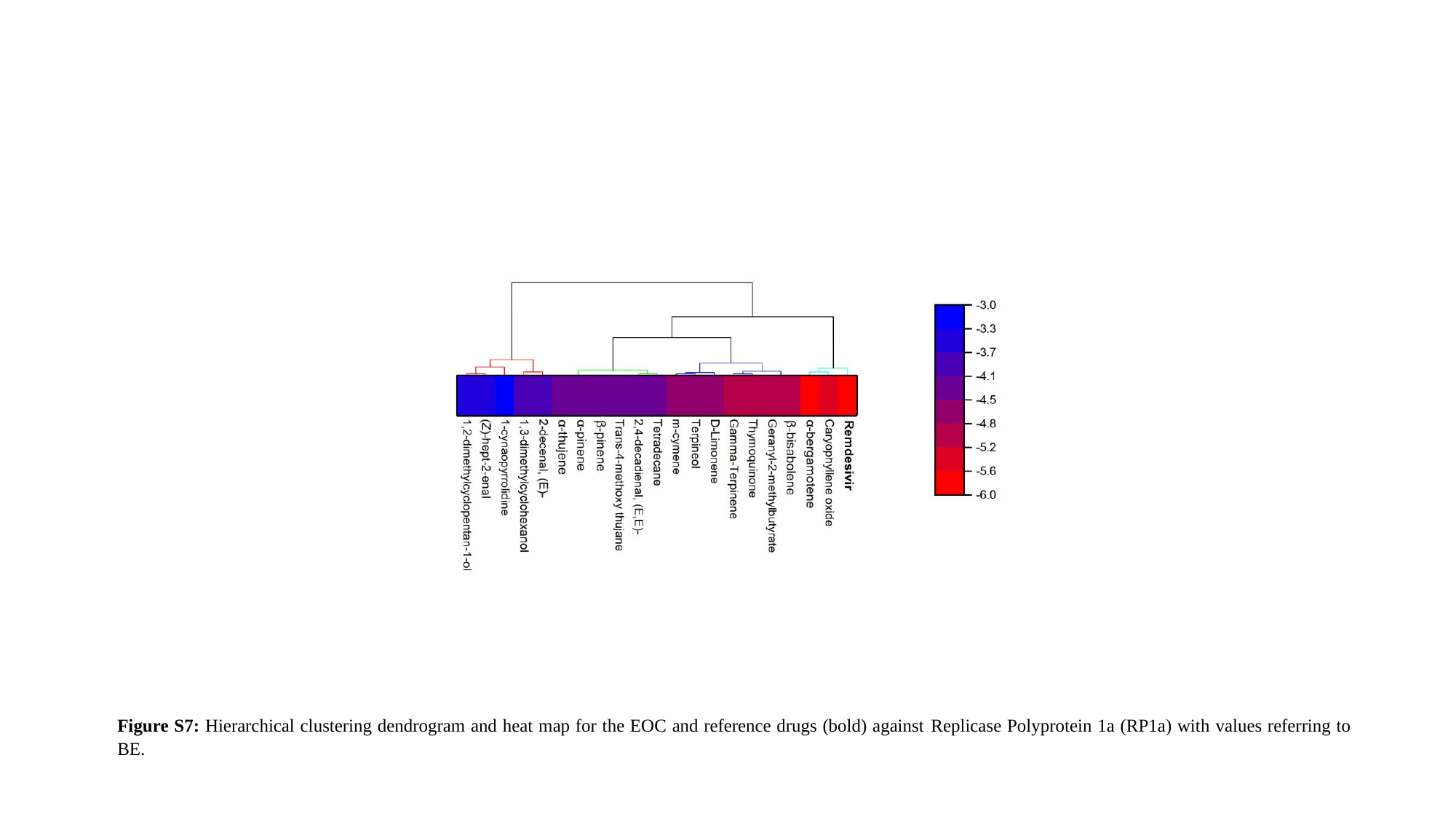

Figure S7: Hierarchical clustering dendrogram and heat map for the EOC and reference drugs (bold) against Replicase Polyprotein 1a (RP1a) with values referring to BE.

## Slide 11
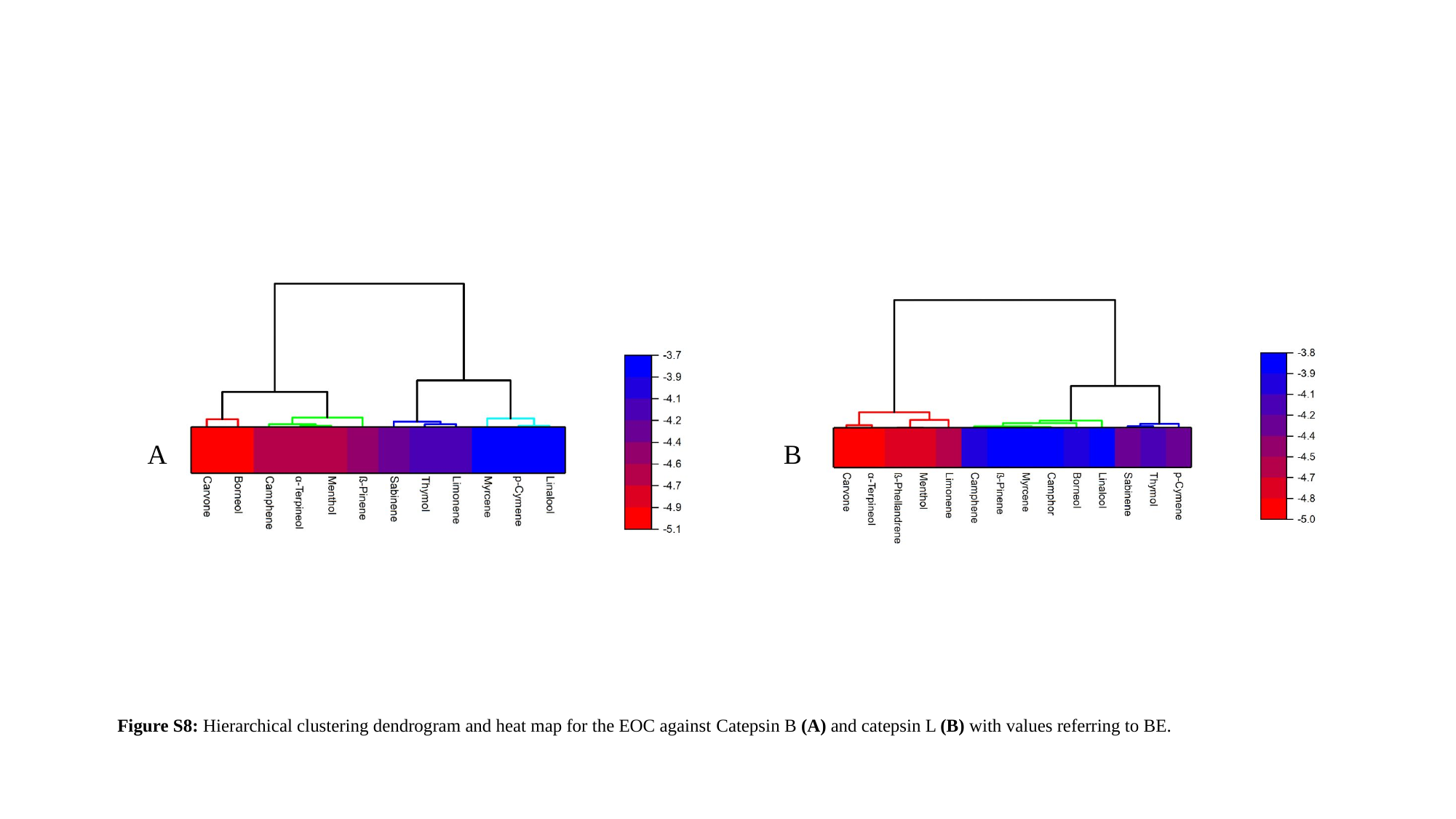

A
B
Figure S8: Hierarchical clustering dendrogram and heat map for the EOC against Catepsin B (A) and catepsin L (B) with values referring to BE.

## Slide 12
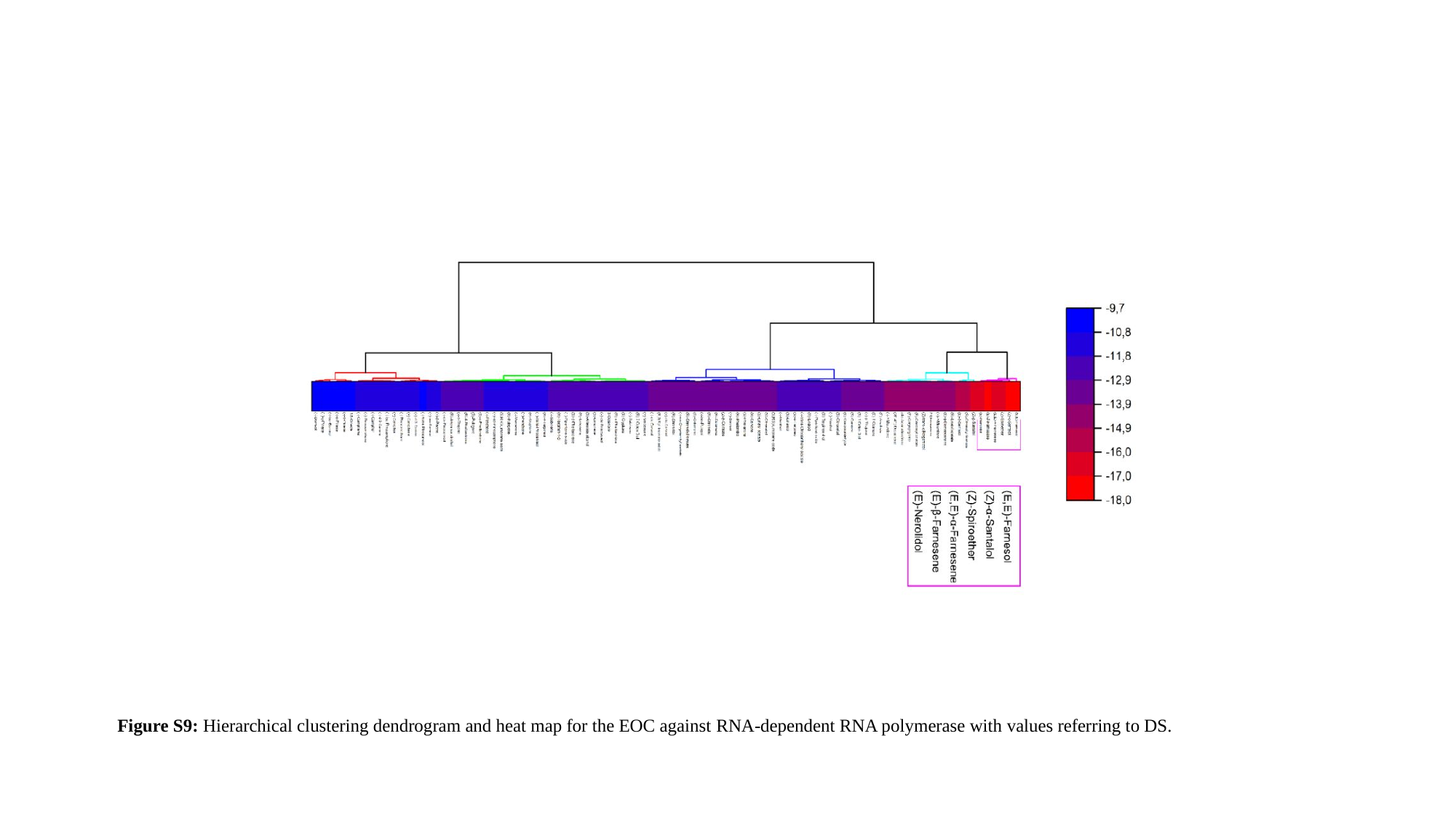

Figure S9: Hierarchical clustering dendrogram and heat map for the EOC against RNA-dependent RNA polymerase with values referring to DS.

## Slide 13
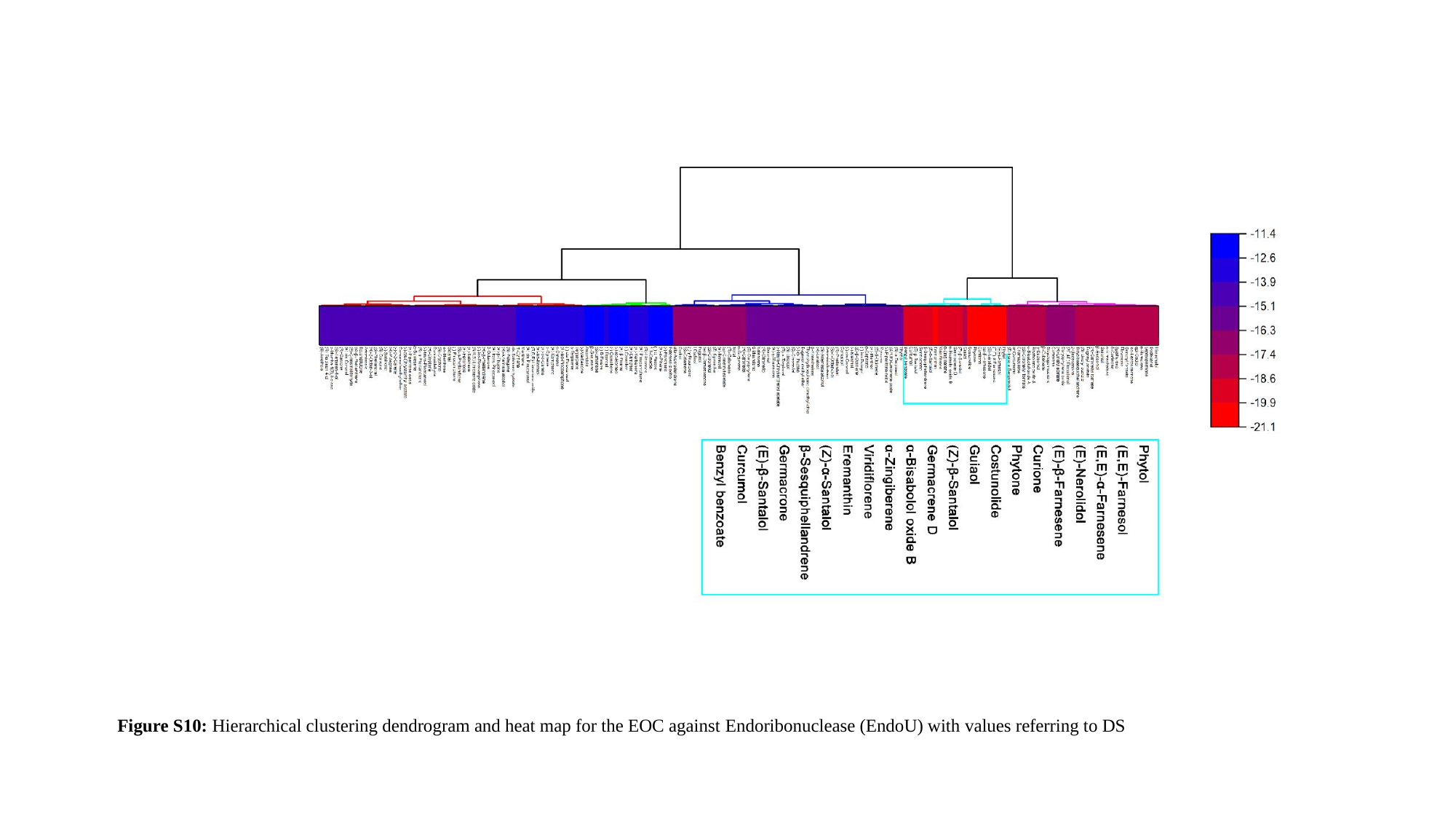

Figure S10: Hierarchical clustering dendrogram and heat map for the EOC against Endoribonuclease (EndoU) with values referring to DS

## Slide 14
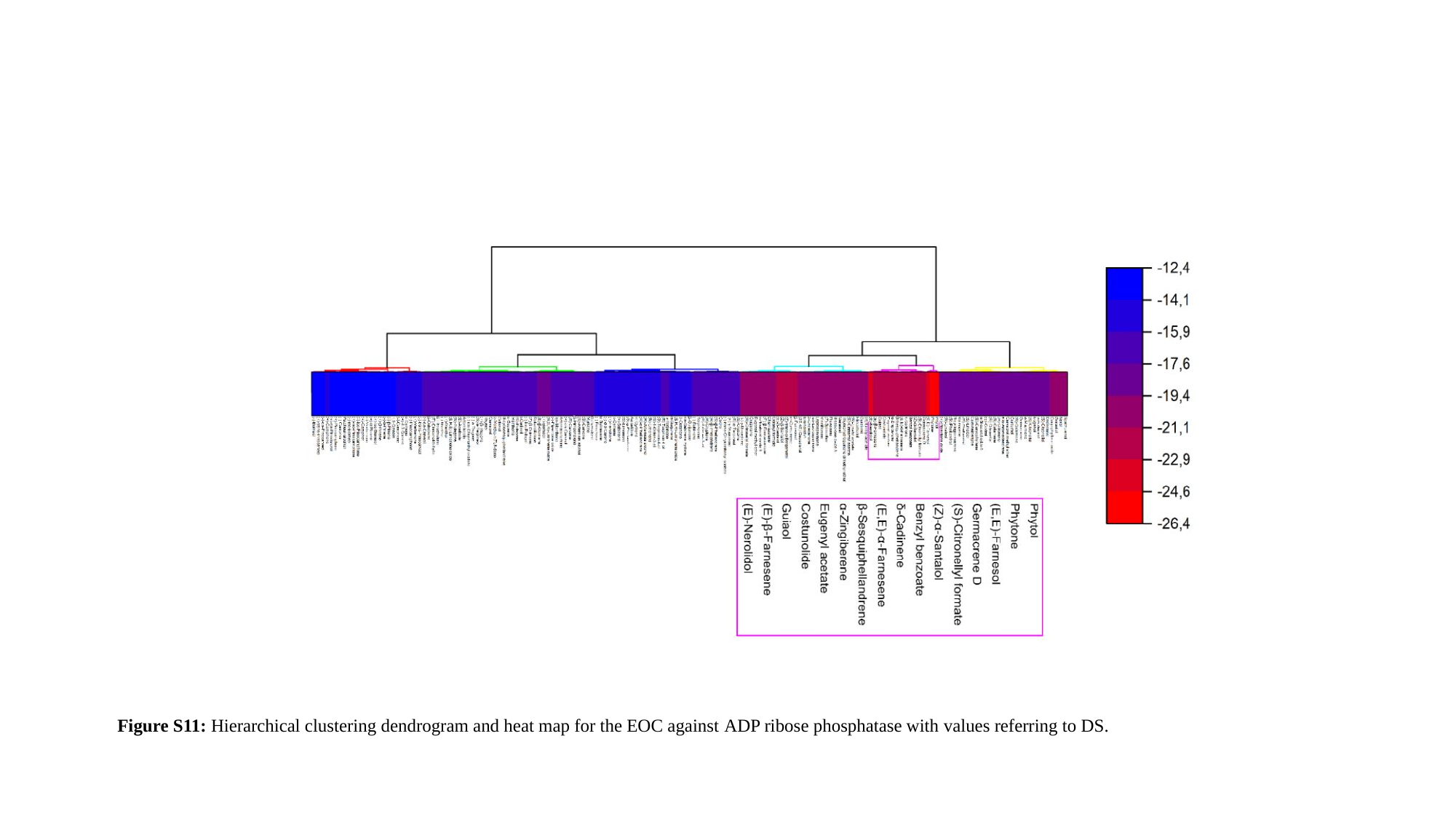

Figure S11: Hierarchical clustering dendrogram and heat map for the EOC against ADP ribose phosphatase with values referring to DS.
